# Comparative risk of adverse perinatal outcomes associated with classes of antiretroviral therapy in pregnant women living with HIV: systematic review and meta-analysis

**DOI:** 10.3389/fmed.2024.1323813

**Published:** 2024-02-27

**Authors:** Katharina Beck, Imogen Cowdell, Clara Portwood, Harriet Sexton, Mary Kumarendran, Zoe Brandon, Shona Kirtley, Joris Hemelaar

**Affiliations:** ^1^National Perinatal Epidemiology Unit, Infectious Disease Epidemiology Unit, Nuffield Department of Population Health, University of Oxford, Oxford, United Kingdom; ^2^Nuffield Department of Orthopaedics, Rheumatology and Musculoskeletal Sciences, Centre for Statistics in Medicine, University of Oxford, Oxford, United Kingdom

**Keywords:** HIV, antiretroviral therapy, protease inhibitor, integrase inhibitor, perinatal outcome, preterm birth, small for gestational age, low birthweight

## Abstract

**Background:**

Integrase strand transfer inhibitor (INSTI) dolutegravir (DTG)-based antiretroviral therapy (ART) is recommended by World Health Organisation as preferred first-line regimen in pregnant women living with human immunodeficiency virus (HIV) (WLHIV). Non-nucleoside reverse transfer inhibitor (NNRTI)-based ART and protease inhibitor (PI)-based ART are designated as alternative regimens. The impact of different ART regimens on perinatal outcomes is uncertain. We aimed to assess the comparative risk of adverse perinatal outcomes in WLHIV receiving different classes of ART.

**Materials and methods:**

A systematic literature review was conducted by searching PubMed, CINAHL, Global Health, and EMBASE for studies published between Jan 1, 1980, and July 14, 2023. We included studies reporting on the association of pregnant WLHIV receiving different classes of ART with 11 perinatal outcomes: preterm birth (PTB), very PTB, spontaneous PTB, low birthweight (LBW), very LBW, term LBW, preterm LBW, small for gestational age (SGA), very SGA (VSGA), stillbirth, and neonatal death. Pairwise random-effects meta-analyses compared the risk of each adverse perinatal outcome among WLHIV receiving INSTI-ART, NNRTI-ART, PI-ART, and nucleoside reverse transfer inhibitor (NRTI)-based ART, and compared specific “third drugs” from different ART classes. Subgroup and sensitivity analyses were conducted based on country income status and study quality.

**Results:**

Thirty cohort studies published in 2006–2022, including 222,312 pregnant women, met the eligibility criteria. Random-effects meta-analyses found no evidence that INSTI-ART is associated with adverse perinatal outcomes compared to NNRTI-ART and PI-ART. We found that PI-ART is associated with a significantly increased risk of SGA (RR 1.28, 95% confidence interval (95% CI) [1.09, 1.51], *p* = 0.003) and VSGA (RR 1.41, 95% CI [1.08, 1.83], *p* = 0.011), compared to NNRTI-ART. Specifically, lopinavir/ritonavir (LPV/r) was associated with an increased risk of SGA (RR 1.40, 95% CI [1.18, 1.65], *p* = 0.003) and VSGA (RR 1.84, 95% CI [1.37, 2.45], *p* = 0.002), compared to efavirenz, but not compared to nevirapine. We found no evidence that any class of ART or specific “third drug” was associated with an increased risk of PTB.

**Conclusion:**

Our findings support the recommendation of INSTI-ART as first-line ART regimen for use in pregnant WLHIV. However, the increased risks of SGA and VGSA associated with PI-ART, compared to NNRTI-ART, may impact choice of second- and third-line ART regimens in pregnancy.

**Systematic review registration**: https://www.crd.york.ac.uk/prospero/, identifier CRD42021248987.

## Introduction

In 2022, 39 million people were living with human immunodeficiency virus (HIV) worldwide, including 15.7 million women of childbearing age (1). An estimated 1.3 million women living with HIV (WLHIV) are pregnant each year, with 90% of these women residing in sub-Saharan Africa (1). Sub-Saharan Africa also has the highest rates of neonatal and child mortality (2). Globally, preterm birth (PTB) is the most important cause of neonatal and child mortality and morbidity (3). Babies born small for gestational age (SGA) contribute to 21.9% of neonatal deaths in low-income and middle-income countries (LMICs) (4). PTB and SGA are both causes of low birthweight (LBW), an outcome commonly used in LMICs when gestational age is uncertain (5). The United Nations’ Sustainable Development Goal 3 (SDG3) target 3.2 aspires to decrease neonatal and under-5 mortality to 12 and 25 per 1,000 live births, respectively, by 2030 (6). However, the vast majority of countries in sub-Saharan Africa are not on track to reach these goals (2). There is therefore an urgent need to address adverse perinatal outcomes that contribute to neonatal and child mortality in this region.

Pregnancies in untreated WLHIV are associated with an increased risk of adverse perinatal outcomes, including PTB, LBW, SGA, and stillbirth, compared to HIV-negative women (7). Antiretroviral therapy (ART, i.e., triple drug therapy) is crucial for WLHIV to improve maternal health and to reduce perinatal HIV transmission. In the past, preconception ART was initiated for maternal reasons (i.e., low CD4 count), whereas antenatal ART was initiated for either prevention of vertical HIV transmission (at high CD4 counts) or for maternal reasons (low CD4 count). In 2013 World Health Organization (WHO) recommended that all pregnant WLHIV should receive ART, irrespective of CD4 counts (8). This led to an increase in the global proportion of WLHIV receiving ART during pregnancy, reaching 81% in 2021 (1). Since 2015, WHO recommend that all people living with HIV should initiate lifelong ART, including pregnant women (9). This led to an increase in the proportion of pregnant WLHIV in sub-Saharan Africa who received ART at the time of conception, from 8% in 2010 to 56% in 2020 (10). However, pregnant WLHIV receiving ART remain at increased risk of PTB, spontaneous PTB, LBW, term LBW, SGA, and very SGA (VSGA), compared with HIV-negative women (11). The question as to whether different ART regimens are associated with different risks of adverse perinatal outcomes has long been controversial, with conflicting data reported (12, 13). In particular, protease inhibitors (PIs), specifically lopinavir/ritonavir (LPV/r), have been associated with an increased risk of PTB in some studies (14–16), but not in others (17–19).

ART consists of a backbone of two nucleoside reverse transcriptase inhibitors (NRTIs) combined with a “third drug” of any class, including integrase strand transfer inhibitors (INSTIs), non-nucleoside reverse transcriptase inhibitors (NNRTIs), PIs, and NRTIs. WHO currently recommends INSTI dolutegravir(DTG)-based ART as preferred first-line regimen for adults, including pregnant women (20). NNRTI efavirenz(EFV)-based ART is an alternative first-line regimen. ART containing PIs, including LPV/r, atazanavir/ritonavir (ATV/r), or darunavir/ritonavir (DRV/r), are designated as second-line or third-line regimens (20). US guidelines recommend DTG-based, or DRV/r-based ART as preferred regimens in pregnancy, with raltegravir(RAL)-based, ATV/r-based, EFV-based or rilpivirin(RPV)-based ART as an alternative regimen (21). European guidelines recommend DTG-based or RAL-based ART or DRV/r-based ART in pregnancy, with EFV-based ART or RPV-based ART as alternative regimens (22).

A network meta-analysis of seven randomized controlled trials (RCTs) compared seven mono-, dual- and triple drug regimens initiated during pregnancy (23). Among the four ART (i.e., triple drug) regimens assessed, zidovudine(ZDV)/lamivudine(3TC)/LPV/r was associated with an increased risk of spontaneous PTB (sPTB) compared to zidovudine/lamivudine/abacavir (ZDV/3TC/ABC; a triple NRTI regimen which is no longer recommended) (24), but no other significant differences in perinatal outcomes between the ART regimens assessed were found. Recent RCTs of ART regimens initiated during pregnancy showed that DTG-based ART had superior virological efficacy compared to EFV-based ART (25, 26). Among regimens with the same backbone, no differences in composite perinatal outcomes were found between DTG-based ART and EFV-based ART, although there was an increase in neonatal death (NND) associated with EFV-based ART (25, 26). A further RCT showed that RAL-based ART also had superior virological efficacy compared to EFV-based ART and no differences in adverse perinatal outcomes were observed (27).

As the number of pregnant WLHIV receiving ART increases, understanding the impact of different ART regimens on perinatal outcomes is crucial. Antiretroviral treatment guidelines cite limited available data concerning pregnancy outcomes associated with antiretroviral drugs (20–22). Few RCTs of ART regimens in pregnancy have been conducted, which enrolled relatively small numbers of women and ART was initiated during the second half of pregnancy, thereby limiting exposure to ART and detection of perinatal outcomes. Observational studies provide important complimentary data, overcome some of the limitations of RCTs, and may provide a more accurate representation of ART regimens, timings of ART initiation, and pregnancy outcomes experienced by pregnant women in the real world. In order to fill this evidence gap, we conducted a systematic review and meta-analysis of observational studies to assess the comparative risk of a range of adverse perinatal outcomes associated with WLHIV receiving INSTI-ART, NNRTI-ART, PI-ART, and NRTI-ART, as well as specific “third drugs” from different ART classes.

## Methods

### Search strategy

We developed a systematic review and meta-analyses protocol based on the Cochrane guidelines (28). A comprehensive literature search strategy, developed by a specialist librarian (SK), was adapted to four electronic literature databases (PubMed, CINAHL (Ebscohost), Global Health (Ovid), EMBASE (Ovid)) to search for studies published between Jan 1, 1980 and July 14, 2023. Free text and controlled vocabulary search terms for “pregnancy outcomes,” “HIV,” and “antiretroviral drugs” were used. No methodological, country, or language filters were applied, and both full-text articles and abstracts were considered. Full search terms are detailed in Supplementary Appendix 1. Retrieved articles were imported into EndNote reference manager (EndNote X20; Clarivate Analytics, Philadelphia, Pennsylvania, United States) and deduplicated. Reference lists of included studies were assessed for additional relevant studies.

The systematic review is registered online (PROSPERO, CRD42021248987) and reported as per the Preferred Reporting Items for Systematic Reviews and Meta-Analyses (PRISMA) guideline (Supplementary Checklist S1) (29).

### Eligibility criteria

Studies were eligible if they included information on the association of pregnant WLHIV receiving different classes of ART with predefined perinatal outcomes. Inclusion criteria were study design (observational studies, i.e., cohort and case control studies), population (pregnant WLHIV), exposure (INSTI-ART, NNRTI-ART, PI-ART, or NRTI-ART) and comparator (different class of ART than the “exposure” group, i.e., INSTI-ART, NNRTI-ART, PI-ART, or NRTI-ART). ART was defined as antiretroviral triple drug therapy. INSTI-ART, NNRTI-ART, PI-ART, and NRTI-ART regimens were defined as two backbone drugs plus any type of INSTI, NNRTI, PI, or NRTI as a “third drug.” Studies were not included if additional treatment was received by one exposure/comparator group only (e.g., anti-tuberculosis treatment), or if less than 95% of WLHIV in an exposure or comparator group conformed to the exposure/comparator definition (e.g., <95% of WLHIV received NNRTI-ART). Preconception and/or antenatal initiation of ART was eligible. Perinatal outcomes assessed were: PTB (birth <37^+0^ weeks gestation) (30); very PTB (VPTB, birth <32^+0^ weeks gestation); sPTB (spontaneous birth <37^+0^ weeks gestation); LBW (<2,500 g) (31); very LBW (VLBW, <1,500 g); SGA (birthweight for gestational age < 10th centile) or very SGA (VSGA, birthweight for gestational age < 3rd centile) according to the reference chart used at the study site (4), stillbirth (delivery of an infant without any signs of life with birthweight ≥1,000 g or gestational age ≥ 24^+0^ weeks, or body length ≥ 35 cm) (32); and NND (death of an infant in the first 28 days of life) (32). Each perinatal outcome was analyzed as a separate outcome, irrespective of potential overlap between different outcomes (e.g., PTB and LBW). Data for term LBW and preterm LBW were sought, but no relevant data was found.

### Study selection

Titles and abstracts of studies retrieved by the literature searches were screened by at least two independent investigators (KB, IC, CP, HS, MK, and ZB) to identify potentially relevant articles. Full text articles of relevant citations were obtained and assessed against the eligibility criteria. Studies were not included if outcomes were not defined or differed from our definitions. If a cohort was reported more than once, the most recent and complete data for each ART comparison and outcome was included. Ambiguities or disagreements regarding inclusion of studies were resolved through discussion with the senior investigator (JH).

### Data extraction

From eligible studies data was extracted regarding study and population characteristics, ART exposures and perinatal outcomes by at least two investigators (KB, IC, CP, HS, MK, and ZB). Unadjusted perinatal outcome data according to class of ART exposure (e.g., NNRTI-ART vs. PI-ART), i.e., outcome frequencies according to ART class exposures, were collected.

In addition, reported unadjusted and adjusted risk ratios (RR) and odds ratios (OR) and 95% confidence intervals (CIs) were also extracted from each publication. If reported, perinatal outcome data according to specific “third drugs” from different classes (e.g., EFV-based ART vs. LPV/r-based ART) were also collected. In addition, methods used to adjust for confounders, including regression analysis, risk factor analysis, and matching (Supplementary Appendix 2) were extracted. Ambiguities or disagreements were resolved through discussion with the senior investigator (JH).

### Quality assessment

The quality of individual studies was assessed using an adapted version of the Newcastle-Ottawa Scale by at least two investigators (KB, IC, CP, HS, MK, and ZB), and reviewed by the senior investigator (JH). Quality assessment criteria were: Selection of study participants (maximum 4 points), Comparability of comparator groups (maximum 2 points), and Assessment of outcomes of interest, including methods to assess gestational age at birth (maximum 3 points). Studies were classified as “good,” “average,” or “poor” quality according to predefined criteria (Supplementary Appendix 2).

### Statistical analysis

Risks of adverse perinatal outcomes were compared between WLHIV receiving INSTI-ART, NNRTI-ART, PI-ART, and NRTI-ART. All analyses were conducted based on frequencies of perinatal outcomes among WLHIV receiving different classes of ART, as extracted from included studies. Pairwise meta-analyses using unadjusted perinatal outcome data from individual studies were carried out if two or more studies reported data for the same ART comparison (e.g., NNRTI-ART vs. PI-ART) and perinatal outcome (e.g., PTB), using a random-effects model to calculate a weighted summary effect estimate (RR), 95% CIs, and *p-*values. *p* < 0.05 were considered statistically significant. Meta-analyses were represented in forest plots and the *I*^2^ statistic was used to quantify heterogeneity due to clinical and methodological variability between studies. The degree of heterogeneity was classified as none (<25%), low (25–49%), moderate (50–74%), or high (≥75%). There are differences between high income countries (HICs) and low- and middle-income countries (LMICs), including HIV prevalence, environmental factors, genetics, and healthcare systems, which may impact the association between ART and perinatal outcomes. Study quality may also impact these associations by addressing bias and confounding. Pre-specified subgroup analyses were therefore performed to separately assess the associations of ART classes with perinatal outcomes in HICs and LMICs, and in average and poor quality studies. In addition, the interaction of country income status and study quality with the association between ART classes and perinatal outcomes was tested (Supplementary Appendix 4). Sensitivity analyses were carried out to assess the effect of adjustment for confounders. The Peters’ test was utilized to assess small study effects in meta-analyses containing a minimum of 10 studies. All statistical analyses were done with Stata version 17 (College Station, Texas, United States).

## Results

**Figure 1:**
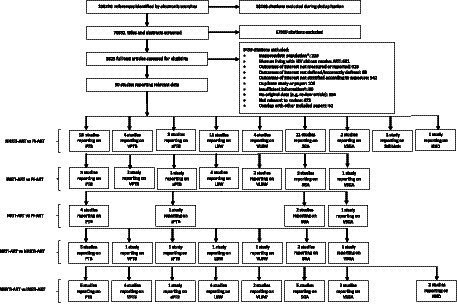
Study selection. *For example, women living with HIV were not pregnant. ^†^For example, paper did not provide relevant outcome data. ART, antiretroviral therapy; HIV, human immunodeficiency virus; INSTI, integrase strand transfer inhibitor; LBW, low birthweight; NND, neonatal death; NNRTI, non-nucleoside reverse transcriptase inhibitor; NRTI, nucleoside reverse transcriptase inhibitor; PI, protease inhibitor, PTB, preterm birth; SGA, small for gestational age; sPTB, spontaneous preterm birth; VLBW, very low birthweight; VPTB, very preterm birth; VSGA, very small for gestational age.

The literature search yielded 108,720 citations, of which 30 studies were included that reported outcome data for pregnant WLHIV receiving INSTI-ART, NNRTI-ART, PI-ART, and NRTI-ART (Figure 1). The perinatal outcomes reported were PTB (22 studies), VPTB (8 studies), sPTB (3 studies), LBW (12 studies), VLBW (6 studies), SGA (15 studies), VSGA (5 studies), stillbirth (1 study), and NND (3 studies).

Characteristics of included studies, published in 2006–2022, are summarized in Table 1 (14–19, 33–56). Ten prospective (33%) and 20 retrospective (67%) cohort studies analyzed data from 222,312 pregnant women in 23 countries (Tables 1, 2). No relevant case–control studies were identified. Sixteen studies (53%) with 48,856 (22%) pregnant women were conducted in HICs, and 14 studies (47%) with 173,456 (78%) pregnant women were conducted in LMICs (Tables 1, 2). Twenty two studies (73%) reported the methods used to determine gestational age, with four (13%) studies using first trimester ultrasound, although none universally, the most accurate method of establishing gestational age (Table 1; Supplementary Appendix 2). Twenty five studies (83%) used methods to assess potential confounding factors. Regression analysis was conducted in 17 studies, risk factor analysis was carried out in 14 studies, and matching of participants was carried out in one study (Table 1; Supplementary Appendix 2). Of the 36 comparisons which were adjusted for covariates in individual studies, none resulted in a change in the statistical significance of the effect estimate (Supplementary Appendix 5). Quality assessments classified 11 studies (37%) as poor quality and 19 (63%) as average quality, with no studies deemed good quality (Table 1; Supplementary Appendix 2). Studies from LMICs had quality ratings (64% average, 36% poor quality) comparable to studies from HICs (63% average, 37% poor quality).

**Table 1:** Characteristics of studies included in the systematic review and meta-analysis.

Study	Country	Country income status	Cohort study design	Recruitment period	Population characteristics*	Method to correct for confounders	Method to estimate gestational age	Quality assessment
Aaron et al. (33)	USA	High	Prospective	1/2000 to 1/2011	First born twin included, 38.3% smoking, 18.0% IDU, urban setting	Regression analysis	LNMP confirmed by second trimester ultrasound	Average
Albert et al (34)	Canada	High	Retrospective	1/1/1997 to 31/1/2018	Twins excluded, women recruited from a provincial surveillance database, 46.1% smoking, 23.3% alcohol use, 26.0% IDU	Risk factor analysis	Ultrasound in first and/or second trimester	Average
Bailey et al. (17)	UK, Ireland, Ukraine, Russia, Belgium, Romania, Spain and Switzerland	High	Retrospective	2008 to 2014	Twins excluded, 6.7% history of IDU in entire cohort	Regression analysis	Ultrasound (unspecified)	Average
Benamor Teixeira et al. (35)	Brazil	Middle	Retrospective	8/2014 to 10/2016	Women recruited from prenatal clinics in urban communities	Risk factor analysis	Ultrasound in first and/or second trimester	Average
Chauhan (36)	India	Middle	Prospective and retrospective	1/2016 to 3/2018	Women receiving ART at a community hospital	None	No description	Poor
Chen et al. (18)	Botswana	Middle	Retrospective	1/5/2009 to 30/4/2011	First born twin included, hospital deliveries, 5.3% alcohol use, 1.7% smoking	Regression analysis, risk factor analysis	LNMP, symphysis-fundal height, or ultrasound (unspecified)	Average
Delicio et al. (37)	Brazil	Middle	Retrospective	2000 to 2015	Women recruited from obstetric clinic serving pregnant women without health insurance from low socioeconomic status, 5.8% alcohol use, 14.3% smoking, 14.3% IDU	Risk factor analysis	No description	Average
Ejigu et al. (38)	Ethiopia	Low	Retrospective	2/2010 to 10/2016	Twins excluded, women recruited from public hospitals and public healthcare centers	Regression analysis, risk factor analysis	Ultrasound, LNMP or fundal height	Average
Ezechi et al. (39)	Nigeria	Middle	Retrospective	7/2004 to 6/2010	Twins included	Regression analysis, risk factor analysis	LNMP	Average
Favarato et al. (16)	UK and Ireland	High	Prospective	2007 to 2015	Twins excluded, 1.7% IDU	Regression analysis	No description	Poor
Favarato et al. (40)	UK and Ireland	High	Prospective	2007 to 2015	Twins excluded, 1.58% IDU	Risk factor analysis	No description	Poor
Floridia et al. (41)	Italy	High	Retrospective	2008 to 2018	Twins excluded, 18.8% smoking, 4.0% recent substance abuse	Risk factor analysis	Ultrasound, LNMP or both	Average
Latham et al. (42)	USA	High	Retrospective	1/2008 to 3/2020	Twins included, 4.8% IDU	None	No description	Poor
López et al. (43)	Spain	High	Prospective	1/2006 to 12/2011	Twins excluded, women recruited in a tertiary hospital, 31.4% smoking, 15.4% history of IDU	Risk factor analysis	First trimester ultrasound and earliest available ultrasound in late gestation	Average
Machado et al. (44)	Brazil	Middle	Prospective	1996 to 2006	Twins excluded, women recruited from a HIV referral center, 21.3% smoking, 5.4% alcohol use, 9% IDU	Regression analysis, risk factor analysis	LNMP or ultrasound	Poor
Patel et al. (45)	USA & Switzerland	High	Prospective	2007 to 2020	17.4% smoking, 8.0% alcohol use	Regression analysis	No description	Average
Piske et al. (46)	Canada	High	Retrospective	2000 to 2012	15.5% smoking	Regression analysis	LNMP	Average
Shapiro et al. (14)	Botswana	Middle	Prospective	7/2006 to 5/2008	Recruited from government run antenatal clinics in urban and rural communities	None	LNMP, ultrasound (in 1^st^, 2^nd^, and 3^rd^ trimester)	Poor
Short et al. (15)	UK	High	Retrospective	1996 to 2010	Twins included, women recruited from a HIV antenatal clinic, urban setting, deliveries in a tertiary hospital, 13.0% smoking	None	No description	Poor
Sibiude et al. (47)	France	High	Retrospective	2005 to 2015	Women enrolled from French Perinatal Cohort	Regression analysis, risk factor analysis	LNMP confirmed by ultrasound	Poor
Sibiude et al. (48)	France	High	Prospective	1/2008 to 12/2017	Women enrolled from French Perinatal Cohort. Twins included	Regression analysis, matching	LNMP confirmed by ultrasound	Average
Snijdewind et al. (49)	Netherlands	High	Retrospective	1/1997 to 2/2015	Twins excluded, women recruited from 26 nationwide sites, 10.8% smoking, 11.7% alcohol use, 0.6% IDU	Risk factor analysis	Early ultrasound or LNMP	Average
Szyld et al. (50)	Argentina, Bahamas, Brazil, and Mexico	Middle	Prospective	1/9/2002 to 1/3/2005	Twins excluded, 9.4% alcohol use, 21.4% smoking, 2.3% IDU	Regression analysis, risk factor analysis	LNMP with/without ultrasound, neonatal assessment (unspecified)	Average
Townsend et al. (19)	UK and Ireland	High	Prospective	1990 to 2005	Twins excluded, 5.0% IDU	Regression analysis	No description	Poor
van der Merwe et al. (51)	South Africa	Middle	Retrospective	10/2004 to 3/2007	Twins excluded, women recruited from HIV referral centers including a tertiary hospital, 3.7% smoking, 3.9% alcohol use	Regression analysis, risk factor analysis	LNMP, ultrasound (unspecified), symphysis-fundal height, neonatal assessment (unspecified)	Poor
Watts et al. (52)	USA and Puerto Rico	High	Retrospective	2007 to 31/10/2010	Twins excluded, 17% smoking,17% smoking, 8.0% alcohol use, 8.0% IDU	Regression analysis	Clinical method (unspecified) and ultrasound (unspecified)	Average
Zash et al. (53)	Botswana	Middle	Retrospective	15/8/2014 to 15/8/2016	Twins excluded, obstetric records extracted at 8 national government hospitals, 6.3% alcohol consumption or smoking	Regression analysis	LNMP confirmed by ultrasound where possible	Average
Zash et al. (54)	Botswana	Middle	Retrospective	14/8/2014 to 15/8/2016	Twins excluded, obstetric records extracted at 8 national government hospitals, 8.3% alcohol consumption or smoking	Regression analysis	LNMP and/or ultrasound (unspecified), or symphysis-fundal height	Average
Zash et al. (55)	Botswana	Middle	Retrospective	1/10/2016 to 31/3/2019	Twins excluded, obstetric records extracted at 8 national government hospitals, 8.7% alcohol consumption or smoking	Regression analysis	LNMP	Average
Zash et al. (56)	Botswana	Middle	Retrospective	8/2014 to 4/2020	Twins excluded, obstetric records extracted at 8 national government hospitals	None	LNMP	Poor

*Details on the inclusion of twins, recruitment center, urban/rural setting, deliveries at home/hospital, smoking, alcohol use, and IDU were sought and reported here if provided by each study. ART, antiretroviral therapy; HIV, human immunodeficiency virus; IDU, illicit drug use; LNMP, last normal menstrual period; NSHPC, National Study of HIV in Pregnancy and Childhood; USA, United States of America; UK, United Kingdom.

**Table 2:** Antiretroviral therapies, ART comparisons, and perinatal outcomes reported by studies included in the systematic review and meta-analysis.

Study	Number of women analyzed	Classes of ART regimens	Antiretroviral “third drugs”	Timing of ART initiation	NNRTI-ART vs PI-ART	INSTI-ART vs PI-ART	NRTI-ART vs PI-ART	NRTI-ART vs NNRTI-ART	NNRTI-ART vs INSTI-ART	Perinatal outcomes
Aaron et al. (33)	183	63.9% PI-ART, 21.3% NNRTI-ART, 14.8% NRTI-ART	**PI:** 2.7% APV, 15.8% ATV, 1.1% DRV, 20.8%, LPV/r, 1.1% FPV, 23% NFV, 15.8% RTV, 19.7% unspecified **NNRTI:** 1.1% EFV, 16.9% NVP, 3.3% ETR, 78.7% unspecified, **NRTI:** Unspecified	Unspecified	Yes	No	Yes	Yes	No	SGA, VSGA
Albert et al. (34)	477	77.1% PI-ART, 18.2% NNRTI-ART 4.7% INSTI-ART	**PI:** IDV, NFV, DRV/c (unspecified proportions) **NNRTI:** NVP, EFV, RPV (unspecified proportions) **INSTI:** DTG, RAL, EVG/c (unspecified proportions)	Mixed	Yes	Yes	No	No	Yes	sPTB
Bailey et al. (17)	7,193	90.3% PI-ART, 9.7% NNRTI-ART	**PI:** 92% LPV/r, 8% unspecified **NNRTI:** Unspecified	Antenatal	Yes	No	No	No	No	PTB, SGA
Benamor Teixeira et al. (35)	390	35.6% PI-ART, 45.1% NNRTI-ART 19.4% INSTI-ART	**PI:** 89.7% LPV/r, 10.3% ATV/r **NNRTI:** 100% EFV **INSTI:** 100% RAL	Antenatal	Yes	Yes	No	No	Yes	PTB, LBW, SGA
Chauhan et al. (36)	87	7.6% PI-ART, 92.4% NNRTI-ART	**PI:** 100% ATV/r **NNRTI:** 65.4% EFV, 34.6% NVP	Mixed	Yes	No	No	No	No	LBW
Chen et al. (18)	33,148	7.6% PI-ART, 92.4% NNRTI-ART	**PI:** 100% LPV/r **NNRTI:** 100% NVP	Mixed	Yes	No	No	No	No	PTB, SGA
Delicio et al. (37)	787	80.8% PI-ART, 19.2% NNRTI-ART	**PI:** 22.7% NFV, 72.1% LPV/r, 5.2% unspecified **NNRTI:** 97.2% NVP, 2.1% EFV, 0.7% unspecified	Mixed	Yes	No	No	No	No	PTB, LBW, VLBW
Ejigu et al. (38)	1,663	2.2% PI-ART, 97.8% NNRTI-ART	**PI:** Unspecified **NNRTI:** 59.4% EFV 40.6% NVP	Mixed	Yes	No	No	No	No	PTB, LBW, SGA
Ezechi et al. (39)	1,843	6.7% PI-ART, 93.3% NNRTI-ART	**PI:** Unspecified **NNRTI:** Unspecified	Mixed	Yes	No	No	No	No	sPTB
Favarato et al. (16)	6,073	68.0% PI-ART, 32.0% NNRTI-ART	**PI:** 38.0% LPV/r, 62.0% unspecified **NNRTI:** Unspecified	Mixed	Yes	No	No	No	No	PTB, SGA
Favarato et al. (40)	10,434	67.5% PI-ART, 32.5% NNRTI-ART	**PI:** 26.9% ATV/r, 9.4% DRV/r, 63.7% LPV/r **NNRTI:** 38.2% EFV 61.8% NVP	Mixed	Yes	No	No	No	No	Stillbirth
Floridia et al. (41)	794	78.5% PI-ART, 15.4% NNRTI-ART, 6.2% INSTI-ART	**PI:** 46.7% ATV, 43.8% LPV, 7.5% DRV **NNRTI:** 60.6% NVP, 26.3% RPV, 4.1% EFV, 1.6% ETR **INSTI:** 59.2% RAL, 28.6% DTG, 10.2% EVG	Mixed	Yes	Yes	No	No	Yes	PTB, VPTB, LBW, VLBW, SGA
Latham et al. (42)	315	29.5% PI-ART, 20.0% NNRTI-ART, 50.5% INSTI-ART	**PI:** Unspecified **NNRTI:** Unspecified **INSTI:** 28.9% RAL, 41.5% EVG, 26.4% DTG, 3.1% BIC	Antenatal	Yes	Yes	No	No	Yes	PTB, LBW
Lopez et al. (43)	156	67.9% PI-ART, 32.1% NNRTI-ART	**PI:** Unspecified **NNRTI:** Unspecified	Mixed	Yes	No	No	No	No	SGA
Machado et al. (44)	696	68.1% PI-ART, 31.9% NNRTI-ART	**PI:** Unspecified **NNRTI:** 100% NVP	Mixed	Yes	No	No	No	No	PTB, LBW
Patel et al. (45)	1,257	51.6% PI-ART, 19.3% NNRTI-ART 29.0% INSTI-ART	**PI:** 71.5% ATV/r, 28.5% DRV/r **NNRTI:** 100% RPV **INSTI:** 32.9% DTG, 23.6% RAL, 43.6% EVG/c	Mixed	Yes	Yes	No	No	Yes	PTB, VPTB, LBW, VLBW, SGA
Piske et al. (46)	1,635	68.0% PI-ART, 18.9% NNRTI-ART, 13.1% NRTI-ART	**PI:** Unspecified **NNRTI:** Unspecified **NRTI:** Unspecified	Mixed	Yes	No	Yes	Yes	No	PTB
Shapiro et al. (14)	730	37.7% PI-ART, 23.3% NNRTI-ART, 39.0% NRTI-ART	**PI:** 100% LPV/r **NNRTI:** 100% NVP **NRTI:** 100% ABC	Antenatal	Yes	No	Yes	Yes	No	PTB, VPTB, LBW, VLBW
Short et al. (15)	331	40.0% PI-ART, 57.6% NNRTI-ART, 2.4% NRTI-ART	**PI:** 100% Unspecified **NNRTI:** 100% NVP **NRTI:** 100% ABC	Mixed	Yes	No	Yes	Yes	No	PTB
Sibiude et al. (47)	1,597	96.0% PI-ART, 4.0% INSTI-ART	**PI:** 46.4% LPV/r, 34.8% ATV/r, 18.7% DRV **INSTI:** 100% RAL	Preconception	No	Yes	No	No	No	VSGA
Sibiude et al. (48)	808	50.0% PI-ART, 50.0% INSTI-ART	**PI:** 100% DRV/r **INSTI:** 87% RAL, 7.1% DTG, 5.9% EVG	Mixed	No	Yes	No	No	No	PTB
Snijdewind et al. (49)	10,795	66.7% PI-ART, 33.3% NNRTI-ART	**PI:** Unspecified **NNRTI:** Unspecified	Mixed	Yes	No	No	No	No	PTB, VPTB, LBW, VLBW, SGA
Szyld et al. (50)	681	56.2% PI-ART, 43.8% NNRTI-ART	**PI:** Unspecified **NNRTI:** Unspecified	Mixed	Yes	No	No	No	No	PTB, LBW
Townsend et al. (19)	4,939	39.7% PI-ART, 58.0% NNRTI-ART, 2.3% NRTI-ART	**PI:** Unspecified **NNRTI:** Unspecified **NRTI:** Unspecified	Mixed	Yes	No	Yes	Yes	No	PTB, VPTB
van der Merwe et al. (51)	1,630	44.5% PI-ART, 55.4% NNRTI-ART	**PI:** 100% LPV/r **NNRTI:** 71.6% NVP 28.4% EFV	Mixed	Yes	No	No	No	No	PTB, LBW, VLBW, SGA
Watts et al. (52)	1,869	79.0% PI-ART, 9.5% NNRTI-ART, 11.5% NRTI-ART	**PI:** Unspecified **NNRTI:** Unspecified **NRTI:** Unspecified	Mixed	Yes	No	Yes	Yes	No	PTB, sPTB, SGA
Zash et al. (53)	46,267	8.0% PI-ART, 92.0% NNRTI-ART	**PI:** 100% LPV/r **NNRTI:** 53.8% EFV 46.2% NVP	Preconception	Yes	No	No	No	No	PTB, VPTB, SGA, VSGA, NND
Zash et al. (54)	57,005	72.7% NNRTI-ART, 27.3% INSTI-ART	**NNRTI:** 100% EFV **INSTI:** 100% DTG	Antenatal	No	No	No	No	Yes	PTB, SGA, NND
Zash et al. (55)	5,701	77.7% NNRTI-ART, 22.3% INSTI-ART	**NNRTI:** 100% EFV **INSTI:** 100% DTG	Preconception	No	No	No	No	Yes	PTB, VPTB, SGA, VSGA, NND
Zash et al. (56)	22,828	74.6% NNRTI-ART 25.4% INSTI-ART	**NNRTI:** 100% EFV **INSTI:** 100% DTG	Antenatal	No	No	No	No	Yes	VPTB, VSGA

ART, antiretroviral therapy; INSTI, integrase strand transfer inhibitor; NNRTI, non-nucleoside reverse transcriptase inhibitor; NRTI, nucleoside reverse transcriptase inhibitor; PI, protease inhibitor. Protease inhibitors: APV, amprenavir; ATV, atazanavir; DRV, darunavir; FPV, fosamprenavir; IDV, indinavir; LPV, lopinavir; NFV, nelfinavir; RTV, ritonavir; /r, ritonavir boosted; /c, cobicistat boosted. Non-nucleoside reverse transcriptase inhibitors: EFV, efavirenz; ETR, etravirine; NVP, nevirapine; RPV, rilpivirine. Nucleoside reverse transcriptase inhibitors: ABC, abacavir. Integrase strand transfer inhibitors: BIC, bictegravir; DTG, dolutegravir; EVG, elvitegravir; RAL, raltegravir. Perinatal outcomes: LBW, low birthweight; NND, neonatal death; PTB, preterm birth; SGA, small for gestational age; sPTB, spontaneous preterm birth; VLBW, very low birthweight; VPTB, very preterm birth; VSGA, very small for gestational age.

**Figure 2:**
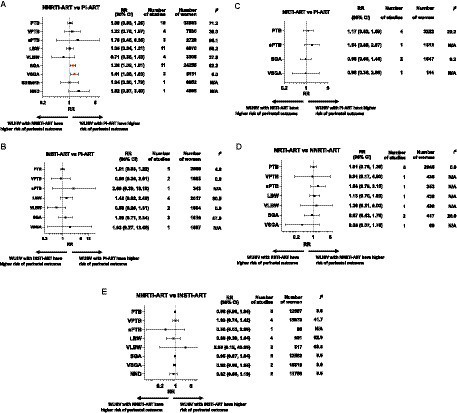
Perinatal outcomes of women living with HIV receiving different ART classes. Random-effects meta-analysis results for perinatal outcomes associated with women living with HIV receiving different classes of ART. Summary effect estimates for associations between each pair of ART classes and each perinatal outcome are shown. Unadjusted risk ratios (RR) and 95% confidence interval (95% CI), *p*-values, numbers of studies and women included in the analysis of each perinatal outcome are displayed. Forest plots of the meta-analyses, based on unadjusted outcome frequencies of perinatal outcomes according to class of ART exposure, can be found in Supplementary Appendix 3. **(A)** NNRTI-ART compared to PI-ART. **(B)** INSTI-ART compared to PI-ART. **(C)** NRTI-ART compared to PI-ART. **(D)** NRTI-ART compared to NNRTI-ART. **(E)** NNRTI-ART compared to INSTI-ART. ART, antiretroviral therapy; INSTI, integrase strand transfer inhibitor; LBW, low birthweight; NND, neonatal death; NNRTI, non-nucleoside reverse transcriptase inhibitor; NRTI, nucleoside reverse transcriptase inhibitor; PI, protease inhibitor; PTB, preterm birth; SGA, small for gestational age; sPTB, spontaneous preterm birth; VLBW, very low birthweight; VPTB, very preterm birth; VSGA, very small for gestational age; WLHIV, women living with HIV.

ART regimens received by WLHIV, ART class comparisons reported, and perinatal outcomes analyzed are shown for each study in Table 2. ART regimes consisted of two backbone drugs plus a “third drug” that was either INSTI, NNRTI, PI, or NRTI. Twenty (67%) studies reported a mixture of preconception and antenatal ART initiation, with others reporting preconception (3 studies; 10%), antenatal (6 studies; 20%), or unspecified (1 study; 3%) ART initiation (Table 2). Random-effects meta-analyses were conducted to compare perinatal outcomes among WLHIV receiving INSTI-ART, NNRTI-ART, PI-ART, and NRTI-ART. The unadjusted summary effect estimates are presented in Figure 2 and the forest plots in Supplementary Appendix 3. Subgroup analyses were carried out according to country income status and study quality (Supplementary Appendix 4).

### NNRTI-ART compared to PI-ART

Twenty five studies, including 134,373 pregnant women, reported on nine perinatal outcomes of WLHIV receiving NNRTI-ART compared to PI-ART (Figure 2A; Table 2). In the analysis of 32,883 WLHIV from 18 studies, PI-ART was not significantly associated with PTB compared to NNRTI-ART (RR 1.08, 95% CI [0.93, 1.25], *p* = 0.332) (Figure 2A). The association remained non-significant in subgroup analyses of studies conducted in LMICs and HICs, and average and poor quality studies (p for interaction = 0.332 and 0.322, respectively) (Supplementary Appendix 4). Specific “third drug” comparisons also did not identify any significant associations (Table 3A).

**Table 3:** Perinatal outcomes of women living with HIV receiving specific “third drugs” of different ART classes.

(A)									
NNRTI-ART vs. PI-ART									
	PTB RR (95%CI) (*p*-value)	VPTB RR (95%CI) (*p*-value)	sPTB RR (95%CI) (*p*-value)	LBW RR (95%CI) (*p*-value)	VLBW RR (95%CI) (*p*-value)	SGA RR (95%CI) (*p*-value)	VSGA RR (95%CI) (*p*-value)	Stillbirth RR (95%CI) (*p*-value)	NND RR (95%CI) (*p*-value)
NVP vs. LPV/r	1.01 (0.69, 1.47) (*p* = 0.961)	1.26 (0.85,1.87) (*p* = 0.241)		1.01 (0.78, 1.31) (*p* = 0.259)	1.22 (0.02, 63.68) (*p* = 0.922)	1.50 (0.55, 4.12) (*p* = 0.922)	1.06 (0.81, 1.40) (*p* = 0.663)	1.11 (0.58, 2.12) (*p* = 0.742)	1.43 (0.74, 2.76) (*p* = 0.287)
NVP vs. ATV/r	0.81 (0.35, 1.86) (*p* = 0.693)			0.88 (0.44, 1.77) (*p* = 0.720)				0.60 (0.24, 1.49) (*p* = 0.269)	
NVP vs. NFV	0.87 (0.56, 1.35) (*p* = 0.527)			0.90 (0.55, 1.46) (*p* = 0.661)					
EFV vs. LPV/r	0.64 (0.18, 2.36) (*p* = 0.319)	1.66 (1.10, 2.50) (*p* = 0.160)		0.48 (0.34, 0.66) (*p* < 0.001)	0.64 (0.17, 2.44) (*p* = 0.513)	1.40 (1.18, 1.65) (*p* = 0.003)	1.84 (1.37, 2.45) (*p* = 0.002)	1.49 (0.62, 3.56) (*p* = 0.370)	2.42 (1.22, 4.80) (*p* = 0.015)
EFV vs. ATV/r				0.84 (0.26, 2.75) (*p* = 0.776)				0.80 (0.27, 2.36) (*p* = 0.683)	
EFV vs. DRV/r								0.98 (0.25, 3.88) (*p* = 0.971)	

(B)									
INSTI-ART vs. PI-ART									
	PTB RR (95%CI) (*p*-value)	VPTB RR (95%CI) (*p*-value)	sPTB RR (95%CI) (*p*-value)	LBW RR (95%CI) (*p*-value)	VLBW RR (95%CI) (*p*-value)	SGA RR (95%CI) (*p*-value)	VSGA RR (95%CI) (*p*-value)	Stillbirth RR (95%CI) (*p*-value)	NND RR (95%CI) (*p*-value)
DTG vs. ATV/r	0.88 (0.56, 1.39) (*p* = 0.581)	0.45 (0.13, 1.52) (*p* = 0.200)		0.97 (0.62, 1.52) (*p* = 0.132)	0.36 (0.12, 1.12) (*p* = 0.078)	1.00 (0.59, 1.70) (*p* = 0.949)			
DTG vs. DRV/r	0.84 (0.49, 1.44) (*p* = 0.533)	0.65 (0.17, 2.54) (*p* = 0.535)		0.88 (0.52, 1.49) (*p* = 0.846)	0.26 (0.05, 1.32) (*p* = 0.103)	0.86 (0.46, 1.62) (*p* = 0.651)			
RAL vs. LPV/r							2.15 (0.30, 15.39) (*p* = 0.447)		
RAL vs. ATV/r	0.97 (0.56, 1.68) (*p* = 0.912)	2.81 (0.16, 48.69) (*p* = 0.478)		0.99 (0.59, 1.67) (*p* = 0.909)	2.81 (0.16, 48.69) (*p* = 0.478)	1.08 (0.57, 2.02) (*p* = 0.865)	1.52 (0.21, 11.19) (*p* = 0.680)		
RAL vs. DRV/r	0.93 (0.50, 1.72) (*p* = 0.816)	4.21 (0.23, 77.32) (*p* = 0.270)		0.90 (0.50, 1.62) (*p* = 0.718)	2.34 (0.11, 48.20) (*p* = 0.582)	0.93 (0.45, 1.90) (*p* = 0.842)	2.17 (0.29, 16.18) (*p* = 0.451)		
EVG/c vs. ATV/r	0.83 (0.56, 1.24) (p = 0.370)	0.60 (0.18, 2.02) (*p* = 0.410)		1.17 (0.75, 1.81) (*p* = 0.488)	0.48 (0.15, 1.49) (*p* = 0.204)	1.17 (0.70, 1.95) (*p* = 0.548)			
EVG/c vs. DRV/r	0.80 (0.49, 1.30) (*p* = 0.367)	0.86 (0.22, 3.38) (*p* = 0.525)		1.05 (0.63, 1.78) (*p* = 0.841)	0.34 (0.07, 1.75) (*p* = 0.198)	1.01 (0.55, 1.86) (*p* = 0.972)			

(C)									
NRTI-ART vs. NNRTI-ART									
	PTB RR (95%CI) (*p*-value)	VPTB RR (95%CI) (*p*-value)	sPTB RR (95%CI) (*p*-value)	LBW RR (95%CI) (*p*-value)	VLBW RR (95%CI) (*p*-value)	SGA RR (95%CI) (*p*-value)	VSGA RR (95%CI) (*p*-value)	Stillbirth RR (95%CI) (*p*-value)	NND RR (95%CI) (*p*-value)
ABC vs. NVP	1.43 (0.10, 21.18) (*p* = 0.793)								

(D)									
NNRTI-ART vs INSTI-ART									
	PTB RR (95%CI) (*p*-value)	VPTB RR (95%CI) (*p*-value)	sPTB RR (95%CI) (*p*-value)	LBW RR (95%CI) (*p*-value)	VLBW RR (95%CI) (*p*-value)	SGA RR (95%CI) (*p*-value)	VSGA RR (95%CI) (*p*-value)	Stillbirth RR (95%CI) (*p*-value)	NND RR (95%CI) (*p*-value)
EFV vs. DTG	0.97 (0.89, 1.06) (*p* = 0.614)	0.97 (0.80, 1.19) (*p* = 0.789)				0.94 (0.86, 1.03) (*p* = 0.158)	0.92 (0.80, 1.05) (*p* = 0.223)		0.92 (0.56, 1.51) (*p* = 0.753)
EFV vs. RAL	0.87 (0.35, 2.16) (*p* = 0.764)			0.50 (0.19, 1.27) (*p* = 0.143)		1.70 (0.76, 3.81) (*p* = 0.198)			
RPV vs DTG	1.31 (0.78, 2.19) (*p* = 0.309)	9.08 (1.03, 80.32) (*p* = 0.047)		1.33 (0.77, 2.32) (*p* = 0.311)	11.36 (1.34, 96.03) (*p* = 0.026)	1.45 (0.77, 2.71) (*p* = 0.252)			

Random-effects meta-analysis results for perinatal outcomes associated with women living with HIV receiving specific “third drugs” of different ART classes. Summary effect estimates for associations between each pair of specific “third drugs” and each perinatal outcome, based on unadjusted outcome frequencies of perinatal outcomes, are shown. **(A)** NNRTI-ART compared to PI-ART, **(B)** INSTI-ART compared to PI-ART, **(C)** NRTI-ART compared to NNRTI-ART, and **(D)** NNRTI-ART compared to INSTI-ART. There was no data comparing specific NRTI and PI “third drugs.” Risk ratios (RR), 95% confidence interval (95% CI), and p values are displayed. A RR > 1 indicates increased risk of a perinatal outcome associated with the second-mentioned drug compared to the first-mentioned drug. For example, in **(A)**, LPV/r-ART is associated with an increased risk of SGA compared to EFV-ART (RR 1.40, 95%CI 1.18–1.65, *p* = 0.003). ART, antiretroviral therapy; INSTI, integrase strand transfer inhibitor; NNRTI, non-nucleoside reverse transcriptase inhibitor; NRTI, nucleoside reverse transcriptase inhibitor; PI, protease inhibitor. Protease inhibitors: ATV, atazanavir; DRV, darunavir; LPV, lopinavir; NFV, nelfinavir; /r, ritonavir boosted. Non-nucleoside reverse transcriptase inhibitors: EFV, efavirenz; NVP, nevirapine; RPV, rilpivirine. Nucleoside reverse transcriptase inhibitors: ABC, abacavir. Integrase strand transfer inhibitors: DTG, dolutegravir; EVG, elvitegravir; RAL, raltegravir; /c, cobicistat boosted. Perinatal outcomes: LBW, low birthweight; NND, neonatal death; PTB, preterm birth; SGA, small for gestational age; sPTB, spontaneous preterm birth; VLBW, very low birthweight; VPTB, very preterm birth; VSGA, very small for gestational age.

In the analysis of 7,530 WLHIV from four studies, PI-ART was not significantly associated with VPTB compared to NNRTI-ART (RR 1.22, 95% CI [0.76, 1.97], *p* = 0.413) (Figure 2A). However, LPV/r-based ART was significantly associated with VPTB compared to EFV-based ART (RR 1.66, 95%CI [1.10, 2.50], *p* = 0.016), but not NVP-based ART (RR 1.26, 95% CI [0.85, 1.87], *p* = 0.241) (Table 3A).

In three average quality studies containing 2,729 WLHIV, PI-ART was not significantly associated with sPTB compared to NNRTI-ART (RR 1.70 95% CI [0.45, 6.35], *p* = 0.388) (Figure 2A). One study analyzing 847 women from a LMIC reported an increased risk of sPTB for PI-ART compared to NNRTI-ART (RR 5.02, 95% CI [3.62, 6.98], *p* < 0.001) (Supplementary Appendix 4).

In the analysis of 6,870 WLHIV from 11 studies, PI-ART was not significantly associated with LBW compared to NNRTI-ART (RR 1.05, 95% CI [0.84, 1.31], *p* = 0.671) (Figure 2A). However, LPV/r-based ART, but not ATV/r-based ART, was significantly associated with a decreased risk of LBW compared to EFV-based ART (RR 0.48, 95% CI [0.34, 0.66], *p* < 0.001). LPV/r, ATV/r, and NFV were not significantly associated with LBW compared to NVP (Table 3A).

Four studies, including 3,308 WLHIV, reported no significant association of PI-ART with VLBW compared to NNRTI-ART (RR 0.71, 95% CI [0.35, 1.43], *p* = 0.355) (Figure 2A). However, two average quality studies, including 2087 WLHIV, conducted in HICs found that PI-ART was associated with a decreased risk of VLBW compared to NNRTI-ART (RR 0.58, 95% CI [0.34, 0.96], *p* = 0.034) (p for interaction = 0.335) (Supplementary Appendix 4).

In the analysis of 24,255 WLHIV from 11 studies, PI-ART was associated with a significantly increased risk of SGA compared to NNRTI-ART (RR 1.28, 95% CI [1.09, 1.51], *p* = 0.003) (Figure 2A). There was moderate heterogeneity (*I*^2^ 62.0, 95% CI [0.0, 83.8]). In subgroup analysis, 7 studies (16,799 women) in HICs showed an increased risk of SGA (RR 1.20, 95% CI [1.03, 1.40], *p* = 0.018), whereas no difference in SGA was seen in 4 studies in LMICs (RR 1.34 95% CI [0.89, 2.02], *p* = 0.157) (p for interaction = 0.620) (Supplementary Appendix 4). The association remained significant in average quality studies (RR 1.19, 95% CI [1.01, 1.41], *p* = 0.040), but not in poor quality studies (RR 1.51 95% CI [0.91, 2.49], *p* = 0.107) (p for interaction = 0.380) (Supplementary Appendix 4). LPV/r-based ART was significantly associated with SGA compared to EFV-based ART (RR 1.40 95% CI [1.18, 1.65], *p* = 0.003), but not compared to NVP-based ART (RR 1.50 95% CI [0.55, 4.12], *p* = 0.922) (Table 3A).

Similarly, in the analysis of 5,151 women from two average quality studies, PI-ART was associated with a significantly increased risk of VSGA compared to NNRTI-ART (RR 1.41 95% CI [1.08, 1.83], *p* = 0.011) (Figure 2A). This association remained significant in the study from a LMIC (RR 1.37 95% CI [1.05, 1.80], *p* = 0.020), but not in the study conducted in a HIC (RR 2.83 95% CI [0.69, 11.72], *p* = 0.150) (p for interaction = 0.332). LPV/r-based ART was significantly associated with VSGA compared to EFV-based ART (RR 1.84 95% CI [1.37, 2.45], *p* = 0.002), but not compared to NVP-based ART (RR 1.06 95% CI [0.81, 1.40], *p* = 0.663) (Table 3A).

In one poor quality study conducted in a HIC analyzing 6,952 women, PI-ART was not associated with stillbirth compared to NNRTI-ART (RR 1.04 95% CI [0.60, 1.79], *p* = 0.891) (Figure 2A). In one average quality study conducted in an LMIC analyzing 4,495 women, PI-ART was not associated with NND (RR 1.82 95% CI [0.97, 3.40], *p* = 0.063) (Figure 2A).

### INSTI-ART compared to PI-ART

Seven studies, including 5,638 women, reported on seven perinatal outcomes of WLHIV receiving PI-ART compared to INSTI-ART (Figure 2B; Table 2). In the analysis of 2,860 WLHIV from five studies, PI-ART was not significantly associated with PTB compared to INSTI-ART (RR 1.01 95% CI [0.83, 1.22], *p* = 0.941) (Figure 2B).

In the analysis of 1,685 WLHIV from two average quality studies conducted in HICs, PI-ART was not significantly associated with VPTB compared to INSTI-ART (RR 0.85 95% CI [0.36, 2.01], *p* = 0.714) (Figure 2B).

One average quality study conducted in a HIC, including 343 WLHIV, found no significant association of PI-ART with sPTB compared to INSTI-ART (RR 2.66 95% CI [0.39, 18.18], *p* = 0.391) (Figure 2B).

Four studies, including 2,017 WLHIV, reported no significant association of PI-ART with LBW compared to INSTI-ART (RR 1.42 95% CI [0.82, 2.48], *p* = 0.211) (Figure 2B). However, one study, including 202 WLHIV, conducted in a LMIC found that PI-ART was associated with an increased risk of LBW compared to INSTI-ART (RR 2.67, 95% CI [1.03, 6.91], *p* = 0.043) (p for interaction = 0.211) (Supplementary Appendix 4).

Two average quality studies conducted in HICs, including 1,654 WLHIV, reported no significant association of PI-ART with VLBW compared to INSTI-ART (RR 0.58, 95% CI [0.26, 1.31], *p* = 0.190) (Figure 2B).

Three average equality studies, including 1,810 WLHIV, reported no significant association of PI-ART with SGA compared to INSTI-ART (RR 1.29 95% CI [0.71, 2.34], *p* = 0.408) (Figure 2B).

A single poor quality study of 1,587 WLHIV conducted in a HIC reported no significant association of PI-ART with VSGA compared to INSTI-ART (RR 1.93 95% CI [0.27, 13.66], *p* = 0.508) (Figure 2B).

The results for perinatal outcomes of WLHIV receiving PI-ART compared to INSTI-ART were reflected in the analyses of “third drugs,” with ATV/r-, DRV/r- or LPV/r-based ART not significantly associated with any adverse perinatal outcomes when compared to DTG-, RAL- or EVG/c-based ART (Table 3B).

### NRTI-ART compared to PI-ART

Six studies containing 9,687 women reported on four perinatal outcomes in WLHIV receiving NRTI-ART compared to PI-ART (Figure 2C; Table 2). In four studies including 3,222 women, PI-ART was not significantly associated with PTB compared to NRTI-ART (RR 1.17 95% CI [0.82, 1.69], *p* = 0.379). Similarly, there was no significant association of PI-ART with sPTB (RR 1.54 95% CI [0.89, 2.67], *p* = 0.122), SGA (RR 0.98 95% CI [0.66, 1.46], *p* = 0.906) or VSGA (RR 0.98 95% CI [0.36, 2.68], *p* = 0.970), compared to NRTI-ART (Figure 2C). There were no data comparing specific NRTI and PI “third drugs.”

### NRTI-ART compared to NNRTI-ART

Six studies containing 9,687 women reported on four perinatal outcomes in WLHIV receiving NNRTI-ART compared to NRTI-ART (Figure 2D; Table 2). In five studies including 2,946 women, there was no significant association of NNRTI-ART with PTB compared to NRTI-ART (RR 1.01 95% CI [0.76, 1.36], *p* = 0.931) (Figure 2D). Similarly, ABC-based ART was not significantly associated with PTB compared to NVP-based ART (RR 1.43 95% CI [0.10, 21.18], *p* = 0.793) (Table 3C).

In the analysis of 439 women from a single poor quality study from a LMIC, NNRTI-ART was not significantly associated with VPTB compared to NRTI-ART (RR 0.91 95% CI [0.17, 4.90], *p* = 0.910) (Figure 2D).

A single average quality study with 353 women from a HIC found no significant association of NNRTI-ART with sPTB compared to NRTI-ART (RR 1.58 95% CI [0.79, 3.15], *p* = 0.196) (Figure 2D).

In the analysis of 439 women from a single poor quality study conducted in a LMIC, NNRTI-ART was not significantly associated with LBW (RR 1.13 95% CI [0.70, 1.83], *p* = 0.625) or VLBW (RR 1.36 95% CI [0.31, 6.00], *p* = 0.684) compared to NRTI-ART (Figure 2D).

In the analysis of 417 women from a single average quality study from a HIC, NNRTI-ART was not significantly associated with SGA compared to NRTI-ART (RR 0.87, 95% CI [0.43, 1.78], *p* = 0.454) (Figure 2D). Likewise, in the analysis of 66 women from a single average quality study from a HIC, NNRTI-ART was not significantly associated with VSGA compared to NRTI-ART (RR 0.35 95% CI [0.07, 1.76], *p* = 0.201) (Figure 2D).

### NNRTI-ART compared to INSTI-ART

Eight studies including 88,767 women reported on eight perinatal outcomes in WLHIV receiving NNRTI-ART compared to INSTI-ART (Figure 2E; Table 2). In 12,687 women from 6 studies, INSTI-ART was not significantly associated with PTB compared to NNRTI-based ART (RR 0.98, 95% CI [0.90, 1.06], *p* = 0.622) (Figure 2E). Similarly, no specific INSTI drugs (DTG and RAL) were associated with PTB compared to specific NNRTI drugs (EFV and RPV) (Table 3D).

In 15,979 women from four average quality studies, INSTI-ART was not significantly associated with VPTB compared to NNRTI-based ART (RR 1.03, 95% CI [0.74, 1.42], *p* = 0.879) (Figure 2E). However, DTG-based ART was associated with VPTB compared to RPV-based ART (RR 9.08, 95% CI [1.03, 80.32], *p* = 0.047), but not compared to EVF-based ART (RR 0.97, 95% CI [0.80, 1.19], *p* = 0.789) (Table 3D).

In one average quality study from a HIC containing 96 women, INSTI-ART was not associated with sPTB compared to NNRTI-ART (RR 0.36, 95% CI [0.05, 2.60], *p* = 0.312) (Figure 2E).

In the analysis of 931 women from four studies, INSTI-ART was not significantly associated with LBW compared to NNRTI-ART (RR 0.81, 95% CI [0.40, 1.62], *p* = 0.551) (Figure 2E).

In the analysis of 517 women from two studies, INSTI-ART was not significantly associated with VLBW compared to NNRTI-ART (RR 2.88, 95% CI [0.19, 43.09], *p* = 0.443) (Figure 2E). However, DTG-based ART was associated with VLBW compared to RPV-based ART (RR 11.36, 95% CI [1.34, 96.03], *p* = 0.026) (Table 3D).

In the analysis of 12,582 women from five average quality studies, INSTI-ART was not significantly associated with SGA compared to NNRTI-ART (RR 0.95, 95% CI [0.87, 1.04], *p* = 0.268)(Figure 2E). Similarly, DTG-based ART and RAL-based ART were not associated with SGA compared to EFV-based ART (Table 3D).

Furthermore, two average quality studies analyzing 15,519 women in LMICs found that.

INSTI-ART was not significantly associated with VSGA compared to NNRTI-ART (RR 0.92, 95% CI [0.80, 1.05], *p* = 0.223) (Figure 2E).

Two average quality studies conducted in a LMIC including 11,756 women found that INSTI-ART was not significantly associated with NND compared to NNRTI-ART (RR 0.82, 95% CI [0.56, 1.19], *p* = 0.288) (Figure 2E).

## Discussion

Our meta-analysis found no evidence that INSTI-ART is associated with any adverse perinatal outcomes assessed compared to NNRTI-ART and PI-ART. We also found that PI-ART is associated with a significantly increased risk of SGA and VSGA, compared to NNRTI-ART. Specifically, LPV/r was associated with an increased risk of SGA and VSGA, compared to EFV. We found no evidence that any class of ART or specific “third drug” was associated with an increased risk of PTB. Our findings should inform clinical guidelines and support the recommendation of INSTI-ART as first-line ART regimen for use in pregnant WLHIV. However, the increased risks of SGA and VGSA associated with PI-ART, compared to NNRTI-ART, may impact choice of second- and third-line ART regimens in pregnancy.

The lack of association between INSTI-ART and any adverse perinatal outcomes, compared to NNRTI-ART and PI-ART, is reassuring. This confirms the findings from RCTs which showed that, among regimens with the same backbone, no differences in composite perinatal outcomes were found with DTG-based ART or RAL-based ART compared to EFV-based ART, although DTG-based ART was associated with a decrease in NND compared to EFV-based ART (25–27). A regimen containing DTG, emtricitabine (FTC) and tenofovir alafenamide fumarate (TAF) had the lowest rate of composite adverse pregnancy outcomes, compared to DTG/FTC/TDF and EFV/FTC/TDF, and a lower rate of preterm birth compared to EFV/FTC/TDF (26). As both DTG-based ART and RAL-based ART had superior virological efficacy compared to EFV-based ART, these findings support the recommendation of INSTI-based ART as first-line ART regimen for use in pregnant WLHIV (20–22).

The finding that PI-ART is associated with a significantly increased risk of SGA and VSGA, compared to NNRTI-ART, but not compared to INSTI-ART, extends findings from a previous meta-analysis which reported that PI-ART was associated with SGA and VSGA compared to non-PI-ART (57). Furthermore, our findings show that LPV/r is associated with an increased risk of SGA and VSGA, compared to EFV, but not compared to NVP. A previous network meta-analysis of RCTs reported no significant differences in perinatal outcomes in LPV/r-based ART compared to EFV-based ART regimens, which may be due to the small numbers of WLHIV enrolled and/or the initiation of ART late in pregnancy (23). We found no cohort studies comparing the risk of SGA or VSGA for ATV/r or DRV/r, the referred PIs in several guidelines (21, 22), with either EFV or NVP. A previous meta-analysis of cohort studies found no significant differences in perinatal outcomes between ART regimens containing LPV/r, ATV/r, and DRV/r (57), and LPV/r is the only PI analyzed in RCTs conducted in pregnant WLHIV to date (23). Overall, these findings support EFV-based ART in preference of PI-based ART regimens and may impact choice of second- and third-line ART regimen for use in pregnant WLHIV. The increased risk of SGA associated with PI-ART may be a consideration for increased antenatal surveillance of fetal growth to enable timely diagnosis and intervention to improve perinatal outcomes.

We found no evidence that NRTI-ART is associated with any adverse perinatal outcomes compared to NNRTI-ART and PI-ART, and no cohort studies compared NRTI-ART with INSTI-ART. A previous network meta-analysis of RCTs reported that ZDV/3TC/LPV/r was associated with an increased risk of sPTB compared to the NRTI-ART regimen ZDV/3TC/ABC (23, 24). Although NRTI-ART is no longer recommended by most treatment guidelines, NRTI-ART regimens may still be used in settings where recommended ART regimens are not available.

The potential risk of PTB associated with maternal HIV infection and ART has received much attention (12). It is noteworthy and reassuring that we found no evidence that any class of ART or specific “third drug” was associated with an increased risk of PTB. This finding is supported by the largest numbers of studies and WLHIV in our analyses, compared to other perinatal outcomes assessed. However, it remains the case that WLHIV receiving ART are at higher risk of PTB compared to WLHIV receiving ZDV monotherapy and HIV-negative women (11, 58). Our findings indicate that choice of ART regimen does not impact the elevated risk of PTB among pregnant WLHIV and hence other interventions are urgently needed to reduce the burden of PTB among WLHIV.

Our meta-analysis has a number of strengths. To our knowledge, this is the first systematic review and meta-analysis comparing all classes of ART, including INSTI-ART, as well as comparing specific “third drugs” from different ART classes. Our study is the largest to date, assessing a comprehensive range of nine perinatal outcomes in WLHIV receiving different classes of ART, including 222,312 pregnant WLHIV from 30 studies. Our study overcame several methodological limitations of previous studies by conducting quality assessments, subgroup and sensitivity analyses, and assessment of correction for confounders (59–63). In particular, the higher quality studies confirmed our findings in the main analyses. 78% of WLHIV analyzed were from LMICs, lending external validity to our findings. Exposures and outcomes were predefined to minimize selection and misclassification bias and promote consistency across studies. A random-effects meta-analysis model was used to account for different study settings. Where applicable, the Peters’ test confirmed an absence of small study effects.

Studies included in our meta-analysis had a number of limitations. All studies were observational and therefore associated with risks of bias, including indication bias linked to WLHIV receiving second- and third-line regimens being more likely to have failed other regimens. Moreover, indication bias may have played a role in relation to the timing of ART initiation. Prior to the current universal treatment policy, preconception ART was initiated for maternal reasons (i.e., low CD4 count), whereas antenatal ART was initiated for either prevention of vertical HIV transmission (at high CD4 counts) or for maternal reasons (low CD4 count). A recent meta-analysis reported that preconception ART initiation was associated with an increased risk of PTB, but no other outcomes, compared to antenatal ART initiation, which may have impacted some of our analyses (64). Most studies (67%) in our meta-analysis included a mix of preconception and antenatal initiation of ART and in the absence of individual patient data it was not possible for us to compare or stratify outcomes for WLHIV initiating ART preconception and antenatally in these studies. For this reason, we were unable to conduct subgroup analyses according to timing of ART initiation. Chronological bias also may have impacted our results. Included studies recruited WLHIV over the past three decades and over this time there have been overall improvements in nutrition, income level, and medical care, which may have affected results obtained in different time periods, especially since the relatively recent introduction of INSTIs. We could not assess the effect of certain important confounders (e.g., CD4 cell count), because of limited reporting of these confounders in included studies. However, we extensively assessed the methods used to assess potential confounding in each study and found that adjustment for covariates by regression analysis did not result in any changes in the significance of the effect estimates in individual studies. However, residual confounding cannot be excluded. There was no data for the comparison of INSTI-ART with NRTI-ART. The perinatal outcomes VPTB, sPTB, VLBW, VSGA, stillbirth and NND were reported in a limited number of studies (1–4 studies) for each ART class comparison. VPTB, VLBW, and VSGA are subsets of the main outcomes (PTB/LBW/SGA) and these outcomes are therefore not independent of each other. VPTB, VLBW, and VSGA represent more severe outcomes, which occur less frequently and are less frequently reported, but which are associated with higher mortality and morbidity. There were fewer studies reporting perinatal outcomes for ART regimens containing specific “third drugs” and the results from these analyses are therefore less reliable. Some confidence intervals were large, indicating significant uncertainty regarding the true values of some effect estimates and a likelihood that effect estimates may change as more data become available in the future. Our analysis was limited to the “third drugs” in triple drug ART regimens and we did not assess the ART backbone. It is possible that backbone drugs differed between ART classes and drugs compared in our analyses and that our findings in part reflect the backbones used and possible interactions between “third drugs” and backbones (26). Unfortunately, data on perinatal outcomes associated with completely defined ART regimens is very limited and should be improved in future studies. Moreover, no study used a universal first trimester ultrasound, the most accurate method to assess gestational age (65). Imprecise assessment of gestational age may have resulted in misclassification bias for PTB, VPTB, SGA and VSGA. SGA and VSGA were defined according to the charts used at individual study sites, rather than an international reference standard (66), which limits comparability of results from different studies. Finally, differences in populations and settings between studies may have contributed to the heterogeneity observed in some of our analyses.

The mechanisms underlying the association between HIV, ART and adverse perinatal outcomes in general, and the link between PI-ART and SGA/VSGA in particular, remain poorly understood. SGA may be due to fetal growth restriction, which may be secondary to placental dysfunction (67). Placental dysfunction may result from altered placental angiogenesis, maternal or placental vascular malperfusion, or metabolic abnormalities, which have been linked to PI-based ART exposure (68). Pre-eclampsia is an important cause of growth restriction and SGA, but maternal HIV infection does not appear to be associated with an increased risk of pre-eclampsia and evidence regarding ART regimens is inconclusive (69). Given the immunodeficiency associated with HIV infection, an immune mechanism of adverse pregnancy outcomes appears plausible. CD4 depletion and chronic immune activation associated with HIV infection may impact the immunological program of pregnancy (70). Innate immune cells, including innate lymphoid cells, mucosal associated invariant T cells and gamma delta γδ T cells, have been reported to be decimated during early HIV infection and not recover with ART, and may be linked to adverse perinatal outcomes (71–73). WLHIV receiving ART have distinct systemic cytokine profiles throughout pregnancy, which may be associated with SGA (74). A recent review extensively examined the current evidence for the potential effects of PIs on progesterone levels, and effects on placenta and decidua (68). It has been reported that WLHIV receiving PI-ART have lower plasma progesterone levels, which may be due to effects of PIs on placental cytochrome P450 enzymes and/or increase in placental expression of 20-alpha-hydroxysteroid dehydrogenase, which inactivates progesterone (68, 75). In both mouse-models and WLHIV receiving PI, reduced progesterone levels are associated with increased risk of SGA (76). A recent RCT of progesterone supplementation in pregnant WLHIV on ART (mostly NNRTI-ART, only 3% PI-ART) showed that administration of 17-alpha-hydroxyprogesterone had no effect on the primary outcomes of PTB or stillbirth. However, progesterone supplementation was instead associated with a reduction in the risk of VSGA, a finding that requires confirmation in additional studies (77).

Given the limited data available for several ART comparisons and perinatal outcomes, it is clear that more and larger prospective observational pregnancy studies among WLHIV are needed to compare different ART regimens. This is particularly important for new antiretroviral drugs, including long-acting antiretrovirals, such as cabotegravir, dual drug regimens, and monoclonal antibodies, for which very limited data in pregnancy is available (78). Moreover, more data is urgently needed regarding antiretroviral drugs used as part of pre-exposure prophylaxis (PrEP) by pregnant HIV-negative women (79). A full range of perinatal outcomes should be assessed, as it is evident that ART regimens differentially impact distinct perinatal outcomes (80). Long-term follow-up is essential to assess effects of intrauterine ART exposure on growth and neurodevelopment of HIV-exposed uninfected children (81).

ART in pregnancy has important benefits for maternal health, prevention of vertical HIV transmission, and prevention of horizontal HIV transmission (20). It is clear that pregnant WLHIV receiving ART remain at increased risk of adverse perinatal outcomes compared to HIV-negative women (11). Further studies are urgently needed to elucidate the mechanisms underlying the adverse perinatal outcomes and to develop preventative and therapeutic interventions to improve perinatal outcomes of WLHIV.

## Data availability statement

The original contributions presented in the study are included in the article/Supplementary material, further inquiries can be directed to the corresponding author.

## Author contributions

KB: Data curation, Formal analysis, Methodology, Visualization, Writing – original draft. IC: Data curation, Formal analysis, Methodology, Visualization, Writing – original draft. CP: Data curation, Methodology, Writing – review & editing. HS: Data curation, Writing – review & editing. MK: Data curation, Writing – review & editing. ZB: Data curation, Writing – review & editing. SK: Data curation, Writing – review & editing, Methodology. JH: Conceptualization, Data curation, Formal analysis, Investigation, Methodology, Project administration, Resources, Supervision, Visualization, Writing – original draft, Writing – review & editing.
