# Supplementary material for: Comparative risk of adverse perinatal outcomes associated with classes of antiretroviral therapy in pregnant women living with HIV: systematic review and meta-analysis
Source: Front Med (Lausanne). 2024 Feb 27;11:1323813. doi: 10.3389/fmed.2024.1323813 (PMC10927998; doi:10.3389/fmed.2024.1323813)
Supplement: Supplementary file 1 [file Data_Sheet_1.docx]

**Appendix**

**Comparative risk of adverse perinatal outcomes associated with classes of antiretroviral therapy in pregnant women living with HIV: systematic review and meta-analysis.**

**Appendices**

APPENDIX 1 3

Literature search strategies 3

Appendix 2 6

Quality assessment of studies 6

Appendix 2.1 6

Adapted Newcastle-Ottawa quality assessment tool

Appendix 2.2 8

Classification of studies according to quality assessment

Appendix 2.3 9

Quality assessment of studies included in the systematic review and meta-analysis

Appendix 2.4 13

Confounding factors adjusted for in included studies

Appendix 3 16

RANDOM EFFECTS META-ANALYSIS OF RISK OF PERINATAL OUTCOMES ASSOCIATED WITH WOMEN LIVING WITH HIV RECEIVING DIFFERENT CLASSES OF ART 16

Appendix 3.1 16

Women living with HIV receiving NNRTI-ART vs PI-ART

**Appendix 3.2** 21

Women living with HIV receiving INSTI-ART vs PI-ART

**Appendix 3.3** 24

Women living with HIV receiving NRTI-ART vs PI-ART

**Appendix 3.4** 26

Women living with HIV receiving NRTI-ART vs NNRTI-ART

Appendix 3.5 28

Women living with HIV receiving NNRTI-ART vs INSTI-ART

Appendix 4 32

SubGROUP ANALYSES 32

**Appendix 4.1** 32

**Subgroup analysis by country income status**

**Appendix 4.2** 34

**Number of studies and women analysed: country income status**

**Appendix 4.3** 35

**I^2^ values: country income status**

**Appendix 4.4** 37

**Subgroup analysis by country income status: test of subgroup difference (interaction)**

**Appendix 4.5**

**Subgroup analysis by study quality 38**

**Appendix 4.6** 39

**Number of studies and women analysed: quality of studies**

**Appendix 4.7** 40

***I^2^* values: quality of studies**

**Appendix 4.8** 41

**Subgroup analysis by quality of study status: test of subgroup difference (interaction)**

Appendix 5 42

SENSITIVITY ANALYSES 42

## **Appendix 5.1** 42

**Adjusting for confounders in individual studies comparing women living with HIV receiving PI-ART, NNRTI-ART, INSTI-ART and NRTI-ART**

APPENDIX 6 44

PERINATAL OUTCOMES OF WOMEN LIVING WITH HIV RECEIVING SPECIFIC ‘THIRD DRUGS’ OF DIFFERENT ART CLASSES.

**Appendix 6.1** 44

5**Number of studies and women analysed: comparisons of specific drugs from different classes**

**Appendix 6.2** 48

***I^2^* values: comparisons of specific drugs from different classes**

**Appendix 6.3** 52

***I^2^* values: comparisons of different ART classes**

# Appendix 1

# Literature search strategies

Search strategy for “pregnancy outcomes” AND “HIV” OR “ARVs”.

Database and platform: MEDLINE (Ovid MEDLINE® Epub Ahead of Print, In-Process & Other Non-Indexed Citations, Ovid MEDLINE® Daily and Ovid MEDLINE®) 1946 to present (via OVID)

Last search date: 14 July 2023

1. Pregnancy Outcome/ or exp Pregnancy Complications, Infectious/

2. ((pregnancy or gestational or fetal or foetal or obstetric$) adj1 (outcome$ or complication$ or consequence$ or characteristic$ or event$ or result$ or problem$ or morbidit$ or sequelae)).ti,ab.

3. ((labor or labour or birth or delivery or neonate or newborn or "new-born" or "new born") adj1 (outcome$ or complication$ or consequence$ or characteristic$ or event$ or result$ or problem$ or morbidit$ or sequelae)).ti,ab.

4. ((infant or reproductive or prelabour or prelabor or "pre-labour" or "pre-labor" or intrauterine or "intra-uterine") adj1 (outcome$ or complication$ or consequence$ or characteristic$ or event$ or result$ or problem$ or morbidit$ or sequelae)).ti,ab.

5. ((antenatal or "ante-natal" or prenatal or "pre-natal" or perinatal or "peri-natal" or neonatal or "neo-natal" or postnatal or "post-natal") adj1 (outcome$ or complication$ or consequence$ or characteristic$ or event$ or result$ or problem$ or morbidit$ or sequelae)).ti,ab.

6. ((antepartum or "ante-partum" or intrapartum or "intra-partum" or peripartum or "peri-partum" or postpartum or "post-partum") adj1 (outcome$ or complication$ or consequence$ or characteristic$ or event$ or result$ or problem$ or morbidit$ or sequelae)).ti,ab.

7. Premature Birth/ or exp Fetal Membranes, Premature Rupture/ or Obstetric Labor, Premature/ or Infant, Extremely Premature/ or Infant, Premature/

8. (prematurity or "gestational age at birth" or "gestational age at delivery" or PTB or PTBs or VPTB or VPTBs or "pre-terms" or preterms or PTL or PTLs or VPTL or VPTLs or PTD or PTDs or VPTD or VPTDs or PROM or PPROM).ti,ab.

9. (("pre-term" or preterm or premature) adj2 (labour$ or labor$ or infant or deliver$ or birth$)).ti,ab.

10. ((preterm or "pre-term" or premature) adj1 rupture adj3 membrane$).ti,ab.

11. Fetal Growth Retardation/ or Infant, Low Birth Weight/ or Infant, Very Low Birth Weight/ or Infant, Extremely Low Birth Weight/ or Infant, Small for Gestational Age/

12. ((intrauterine or "intra-uterine" or fetal or foetal) adj1 growth adj1 (restrict$ or retardation)).ti,ab.

13. (SGA or SFGA or IUGR or FGR or "small for gestational age" or "small-for-gestational-age" or "small-for-gestational age" or "small for gestation" or "small-for-gestation").ti,ab.

14. (VSGA or "very-small-for-gestational-age" or "very-small-for-gestational age" or SFD or "small for dates" or "small-for-dates" or "weight for dates" or "weight for gestational age" or "weight for age at delivery" or "weight at delivery").ti,ab.

15. ("birthweight for dates" or "birthweight for gestational age" or "birthweight for age at delivery" or "birth weight for dates" or "birth weight for gestational age" or "birth weight for age at delivery" or "birth-weight for dates" or "birth-weight for gestational age" or "birth-weight for age at delivery").ti,ab.

16. (LBW or "low BW" or "low birth weight" or "low birth-weight" or "low-birth weight" or "low-birth-weight" or "low birthweight" or "low-birthweight" or "lower BW" or "lower birth weight" or "lower birth-weight" or "lower-birth weight" or "lower-birth-weight" or "lower birthweight" or "lower-birthweight").ti,ab.

17. ("reduced birth weight" or "reduced birthweight" or "reduced birth-weight" or VLBW or "very-low birthweight" or "very-low birth weight" or "very-low birth-weight" or "very-low-birthweight" or "very-low-birth-weight" or ELBW or "extremely-low birthweight" or "extremely-low birth weight" or "extremely-low birth-weight" or "extremely-low-birthweight" or "extremely-low-birth-weight").ti,ab.

18. Stillbirth/ or Fetal Death/

19. (stillbirth$ or "still birth$" or stillborn$ or "still born$" or abortion$ or miscarriage$).ti,ab.

20. ((pregnancy or gestational or fetal or foetal or obstetric$ or labor or labour or birth) adj1 (death$ or loss$ or demise$ mortalit$)).ti,ab.

21. ((delivery or neonate or newborn or "new-born" or "new born" or infant or reproductive or prelabour or prelabor or "pre-labour" or "pre-labor") adj1 (death$ or loss$ or demise$ mortalit$)).ti,ab.

22. ((intrauterine or "intra-uterine" or antenatal or ante-natal or prenatal or "pre-natal" or perinatal or "peri-natal" or neonatal or "neo-natal" or postnatal or "post-natal" or antepartum or "ante-partum" or intrapartum or "intra-partum" or peripartum or "peri-partum" or postpartum or "post-partum") adj1 (death$ or loss$ or demise$ mortalit$)).ti,ab.

23. 1 or 2 or 3 or 4 or 5 or 6 or 7 or 8 or 9 or 10 or 11 or 12 or 13 or 14 or 15 or 16 or 17 or 18 or 19 or 20 or 21 or 22

24. HIV/ or HIV Seropositivity/ or HIV Infections/ or HIV-2/ or HIV-1/ or AIDS Serodiagnosis/ or Acquired Immunodeficiency Syndrome/ or AIDS Arteritis, Central Nervous System/ or AIDS-Associated Nephropathy/ or AIDS Dementia Complex/ or AIDS-Related Opportunistic Infections/ or Lymphoma, AIDS-Related/

25. (HIV or "HIV/AIDS" or PLHIV or PLWHA or WLHIV or WLWHA or "HIV-1" or "HIV-type-1" or "HTLV III" or "HTLV-III" or "HTLV type III" or "HTLV-type-III" or LAV or "HTLV-III-LAV" or "LAV-HTLV-III" or "HIV-2" or "HIV-type-2" or "HIV-II" or "HTLV-IV" or "LAV-2").ti,ab.

26. ("HIV-positive" or "HIV-1-positive" or "HIV-2-positive" or "HIV-infected" or "HIV-1-infected" or "HIV-type-1-infected" or "HTLV III-infected" or "HTLV-III-infected" or "HTLV type III-infected" or "HTLV-type-III-infected" or "LAV-infected").ti,ab.

27. ("HTLV-III-LAV-infected" or "LAV-HTLV-III-infected" or "HIV-2-infected" or "HIV-type-2-infected" or "HIV-II-infected" or "HTLV-IV-infected" or "LAV-2-infected" or "HIV-infection$" or "HIV-1-infection$" or "HIV-type-1-infection$" or "HTLV III-infection$" or "HTLV-III-infection$" or "HTLV type III-infection$" or "HTLV-type-III-infection$").ti,ab.

28. ("LAV-infection$" or "HTLV-III-LAV-infection$" or "LAV-HTLV-III-infection$" or "HIV-2-infection$" or "HIV-type-2-infection$" or "HIV-II-infection$" or "HTLV-IV-infection$" or "LAV-2-infection$" or "Human Immunodeficiency Virus$" or "Human Immune Deficiency Virus$").ti,ab.

29. ("Human T Cell Lymphotropic Virus Type III" or "Human T-Cell Lymphotropic Virus Type III" or "Human T Lymphotropic Virus Type III" or "Human T-Lymphotropic Virus Type III" or "Human T Lymphotropic Virus Type IV" or "Human T-Lymphotropic Virus Type IV" or "Human T Cell Leukemia Virus Type III" or "Human T-Cell Leukemia Virus Type III").ti,ab.

30. ("Lymphadenopathy-Associated Virus$" or "Lymphadenopathy Associated Virus$" or AIDS or "Acquired Immune Deficiency Syndrome" or "Acquired Immunodeficiency Syndrome").ti,ab.

31. 24 or 25 or 26 or 27 or 28 or 29 or 30

32. exp Anti-HIV Agents/ or exp HIV Fusion Inhibitors/ or exp HIV Integrase Inhibitors/ or exp HIV Protease Inhibitors/ or HIV Reverse Transcriptase/ or Reverse Transcriptase Inhibitors/

33. ((antiretroviral or "anti-retroviral" or antiviral$ or "anti-viral$" or "anti-HIV" or "anti-HIV-1") adj1 (treatment$ or therap$ or regimen$ or drug$ or agent$)).ti,ab.

34. (("anti-HIV-2" or "anti-AIDS") adj1 (treatment$ or therap$ or regimen$ or drug$ or agent$)).ti,ab.

35. (HAART or "HAART-exposed" or "HAART-treated" or "Mega-HAART" or ARV or ARVs or cARV or cARVs or "ARV-exposed" or "ARV-treated" or "combination-ARV" or "combination-ARVs" or "combined-ARV" or "combined-ARVs").ti,ab.

36. (ART or "Multi-ART" or "Triple-ART" or cART or "ART-exposed" or "ART-treated" or "combination-ART" or "combined-ART" or "sc-ART" or "short-course-antiretroviral therap$" or "short-course-anti-retroviral therap$").ti,ab.

37. (combin$ adj (treatment$ or therap$ or regimen$ or drug$ agent$)).ti,ab.

38. (monotherap$ or "mono-therap$" or "dual therap$" or "dual drug therap$" or bitherap$).ti,ab.

39. (PI or PIs or "PI-based" or "boosted-PI" or "PI-containing" or "PI-therap$" or "PI-treatment$" or "PI-regimen$" or "Ritonavir-boosted" or "protease inhibitor$" or NRTI or NRTIs or "NRTI-based" or "NRTI-containing" or "NRTI-therap$" or "NRTI-treatment$" or "NRTI-regimen$" or "nucleoside reverse transcriptase inhibitor$" or "nucleoside analog reverse transcriptase inhibitor$").ti,ab.

40. (NNRTI or NNRTIs or "NNRTI-based" or "NNRTI-containing" or "NNRTI-therap$" or "NNRTI-treatment$" or "NNRTI-regimen$" or "non nucleoside reverse transcriptase inhibitor$" or "non-nucleoside reverse transcriptase inhibitor$" or "nonnucleoside reverse transcriptase inhibitor$" or "non nucleoside analog reverse transcriptase inhibitor$" or "non-nucleoside analog reverse transcriptase inhibitor$" or "nonnucleoside analog reverse transcriptase inhibitor$").ti,ab.

41. (NtRTI or NtRTIs or "NtRTI-based" or "NtRTI-containing" or "NtRTI-therap$" or "NtRTI-treatment$" or "NtRTI-regimen$" or "nucleotide reverse transcriptase inhibitor$" or "nucleotide analog reverse transcriptase inhibitor$" or "fusion inhibitor$" or "CCR5 receptor antagonist$" or "integrase inhibitor$" or "maturation inhibitor$" or "entry inhibitor$").ti,ab.

42. Didanosine/ or Delavirdine/ or Emtricitabine/ or Lamivudine/ or Nevirapine/ or Rilpivirine/ or Stavudine/ or Tenofovir/ or Zidovudine/ or Atazanavir Sulfate/ or Darunavir/ or Ritonavir/ or Lopinavir/ or Nelfinavir/ or Saquinavir/ or Enfuvirtide/ or Maraviroc/ or Raltegravir Potassium/ or Indinavir/ or Zalcitabine/ or Cobicistat/

43. "Efavirenz, Emtricitabine, Tenofovir Disoproxil Fumarate Drug Combination"/ or "Emtricitabine, Rilpivirine, Tenofovir Drug Combination"/ or "Emtricitabine, Tenofovir Disoproxil Fumarate Drug Combination"/ or "Elvitegravir, Cobicistat, Emtricitabine, Tenofovir Disoproxil Fumarate Drug Combination"/

44. (Abacavir or ABC or Didanosine or ddI or Emtricitabine or FTC or Lamivudine or 3TC or Stavudine or d4T or Tenofovir or TFV or TDF or TAF or Zidovudine or AZT or ZDV or Delavirdine or DLV or Efavirenz or EFV or Etravirine or ETR or Nevirapine or NVP or Rilpivirine or RPV or Atazanavir or ATV or "Atazanavir/Ritonavir" or "ATV/r" or Darunavir or DRV or "Darunavir/Ritonavir" or "DRV/r" or Fosamprenavir or FPV or "Fosamprenavir/Ritonavir" or "FPV/r" or temsavir or TMR).ti,ab.

45. (Indinavir or IDV or "Indinavir/Ritonavir" or "IDV/r" or Lopinavir or LPV or "Lopinavir/Ritonavir" or "LPV/r" or Nelfinavir or NFV or "Nelfinavir/Ritonavir" or "NFV/r" or Ritonavir or RTV or Saquinavir or SQV or "Saquinavir/Ritonavir" or "SQV/r" or Tipranavir or TPV or "Tipranavir/Ritonavir" or "TPV/r" or Enfuvirtide or "T-20" or Maraviroc or MVC or Raltegravir or RAL or Elvitegravir or EVG or Zalcitabine or ddC or Combivir or Trizivir or Kaletra or Epzicom or Kivexa or Truvada or Atripla).ti,ab.

46. ("Integrase strand transfer inhibitor" or Dolutegravir or DTG or Tivicay or Isentress or Vitekta or "Formyl peptide receptor 1" or Fuzeon or FPR1 or ENF or Seizentry or Celsentri or Ziagen or Videx or Emtriva or Coviracil or Zerit or Viread or Vemlidy or Retrovir or Azidothymidine or "Diarylpyrimidine analogue" or Rescriptor or Sustiva or Intelence or TMC125 or DAPY or Viramune or Edurant or TMC278).ti,ab.

47. (Reyataz or APV or Agenerase or Prezista or Lexiva or Telzir or Crixivan or ABT-378 or Norvir or Viracept or AG1343 or Invirase or Fortovase or Aptivus or "Rilpivirine plus dolutegravir" or "RPV/DTG" or "Raltegravir plus lamivudine" or "RAL/3TC" or "Abacavir plus lamivudine plus dolutegravir" or "ABC/3TC/DTG" or "Emtricitabine plus tenofovir alafenamide" or "FTC/TAF" or "Emtricitabine plus rilpivirine plus tenofovir alafenamide" or "FTC/RPV/TAF" or "Atazanavir plus cobicistat" or "ATV/COBI" or "Darunavir plus cobicistat" or "DRV/COBI" or Juluca or Dutrebis or Stribild or Triumeq or Odefsey or Complera or Descovy or Genvoya or Evotaz or Prezcobix).ti,ab.

48. (Cobicistat or "cobicistat-boosted" or COBI or Rezolsta or QUAD or Epivir or Temixys or Cimduo or Selzentry or Doravirine or DOR or Pifeltro or "Ibalizumab-uiyk" or Hu5A8 or IBA or Ibalizumab or "TMB-355" or "TNX-355" or Trogarzo or Bictegravir or BIC or "Bictegravir, Emtricitabine, Tenofovir Alafenamide" or "bictegravir sodium/emtricitabine/tenofovir alafenamide fumarate" or "BIC/FTC/TAF" or Biktarvy or "tenofovir alafenamide fumarate" or "darunavir ethanolate, cobicistat, emtricitabine, tenofovir alafenamide fumarate" or "DRV/COBI/FTC/TAF").ti,ab.

49. (Symtuza or "Dolutegravir and lamivudine" or "dolutegravir sodium/lamivudine" or "DTG/3TC" or Dovato or "Doravirine, lamivudine, and tenofovir disoproxil fumarate" or "doravirine/lamivudine/tenofovir disoproxil fumarate" or "DOR/3TC/TDF" or Delstrigo or "Efavirenz, lamivudine, and tenofovir disoproxil fumarate" or "EFV/3TC/TDF" or Symfi or "Symfi Lo" or "Elvitegravir, cobicistat, emtricitabine, and tenofovir alafenamide fumarate" or "elvitegravir/cobicistat/emtricitabine/tenofovir alafenamide fumarate" or "EVG/COBI/FTC/TAF" or Genvoya or "TMC-114" or "TMC114" or Dideoxyinosine or Racivir or Heptovir or Hepitec).ti,ab.

50. (Zerut or Estavudina or Sanilvudine or Apropovir or Stocrin or Zrivada or Aluvia or Aluviran or Pentafuside or Symtuza or "Efavirenz, Lamivudine, Tenofovir Disoproxil Fumarate drug combination" or "Lopinavir-Ritonavir drug combination").ti,ab.

51. (NRTTI or NRTTIs or "NRTTI-based" or "NRTTI-containing" or "NRTTI-therap$" or "NRTTI-treatment$" or "NRTTI-regimen$" or "nucleoside reverse transcriptase translocation inhibitor" or islatravir or ISL or "MK-8591" or "portmanteau inhibitor$" or "capsid inhibitor$" or "CAI" or lenacapavir or "LEN" or "GS-CA1" or leronlimab or albuvirtide or Eviplera or "rilpivirine, emtricitabine and tenofovir disoproxil fumarate" or "attachment inhibitor" or "gp120 attachment inhibitor" or Rukobia or fostemsavir or "FTV" or INSTI or INSTIs or Vocabria or cabotegravir or "CAB" or cabenuva or Tybost or "INSTI-based" or "INSTI-containing" or "INSTI-therap$" or "INSTI-treatment$" or "INSTI-regimen$").ti,ab.

52. 32 or 33 or 34 or 35 or 36 or 37 or 38 or 39 or 40 or 41 or 42 or 43 or 44 or 45 or 46 or 47 or 48 or 49 or 50 or 51

53. 31 or 52

54. 23 and 53

55. limit 54 to yr="2020-2023"

# Appendix 2

# Quality assessment of studies

## **Appendix 2.1**

## **Adapted Newcastle-Ottawa quality assessment tool**

A study can be awarded a maximum of one point (for items indicated with an asterisk) for each numbered criterion within the “Selection” and “Outcome” categories.

**Selection (maximum 4 points)**

1) Representativeness of the exposed cohort.

a) Truly representative of the pregnant population in the community. *

b) Somewhat representative of the pregnant population in the community.

c) Selected group of users, e.g. nurses, volunteers, teenage mothers.

d) No description of the derivation of the cohort.

2) Selection of the comparator cohort.

a) The comparator cohort is drawn from the same community as the exposed cohort. *

b) The comparator cohort is drawn from a different source than the exposed cohort.

c) No description of the derivation of the comparator cohort.

3) Ascertainment of exposure.

a) ART intake monitored as part of study. *

b) ART intake confirmed from secure medical records (e.g. hospital records). *

c) Structured interview-participant reported ART intake.

d) Written self-report.

e) No description.

4) Demonstration that outcome of interest was not present at start of study.

a) Yes. *

b) No.

**Comparability (maximum 2 points)**

1) Comparability of cohorts on the basis of the analysis. In the analysis:

a) Study controls for BMI, smoking, parity, and maternal age. *

b) Study controls for one or more additional factors: e.g. prior history of adverse pregnancy outcome, maternal hypertension, anaemia, illicit drug or alcohol use in pregnancy. *

c) Confounding factors not controlled for.

**Outcome (maximum 3 points)**

1) Ascertainment of outcome.

a) Outcome was confirmed following clinical observation of outcome by clinician, midwife or trained birth attendant. *

b) Medical records. *

c) Self-report.

d) No description.

2) Method used to assess gestational age.

a) Gestational age was determined according to early ultrasound (<14 weeks). *

b) Gestational age was determined by: late ultrasound (≥14 weeks’ gestation) or last normal menstrual period or neonatal assessment, e.g. Ballard score, or a combination of these methods.

c) No description.

3) Follow up of cohorts

a) Complete follow up - all subjects accounted for. *

b) Subjects lost to follow up unlikely to introduce bias, i.e. < 20 % lost to follow up. *

c) Follow up rate < 80% (lost to follow-up > 20%).

d) No description.

## **Appendix 2.2**

## **Classification of studies according to quality assessment**

| Good Quality | 9 points – all requirements met |
| --- | --- |
| Average Quality | 3 points in “Selection” and 3 points in “Outcome” sections |
|  | ≥2 points in the “Selection” and “Outcome” sections, as well as ≥1 point in the “Comparability” section. |
| Poor Quality | < 2 points in the “Selection” and/or “Outcome” sections. |
|  | 2 points in the “Selection” and “Outcome” sections, but no points in the “Comparability” section. |

##

## **Appendix 2.3**

## **Quality assessment of studies included in the systematic review and meta-analysis**

| **Study** | **Representativeness of the exposed cohort** | **Selection of comparator cohort** | **Ascertainment of exposure** | **Demonstration that outcome of interest was not present at start of study** | **Comparability of cohorts on the basis of analysis** | **Ascertainment of outcome** | **Method used to assess gestational age** | **Follow up of cohorts** | **Total quality assessment** |
| --- | --- | --- | --- | --- | --- | --- | --- | --- | --- |
| **Aaron (2012)** | Truly representative* | Same community as exposed cohort* | Medical records* | Yes* | Study controls for one or more additional confounding factors* | Medical records* | LNMP confirmed by 2^nd^ trimester ultrasound | <20% lost to follow up* | Average |
| **Albert (2020)** | Truly representative* | Same community as exposed cohort* | Medical records* | No | Study controls for one or more additional confounding factors* | Medical records* | Ultrasound in first and/or second trimester | Complete follow up* | Average |
| **Bailey (2019)** | Truly representative* | Same community as exposed cohort* | Medical records* | No | Study controls for one or more additional confounding factors* | Clinical observation* | Ultrasound (unspecified) | Complete follow up* | Average |
| **Benamor Teixeria**  **(2020)** | Truly representative* | Same community as exposed cohort* | Medical records* | No | Study controls for one or more additional confounding factors* | Medical records* | Ultrasound (unspecified) | <20% lost to follow up* | Average |
| **Chauhan (2021)** | Truly representative* | Same community as the exposed population* | ART intake monitored as part of study * | No | Confounding factors not controlled for | No description | No description | <20% lost to follow up* | Poor |
| **Chen (2012)** | Truly representative* | Same community as exposed cohort* | Medical records* | No | Study controls for one or more additional confounding factors* | Medical records* | LNMP, symphysis-fundal height, or ultrasound (unspecified) | <20% lost to follow up* | Average |
| **Delicio (2018)** | Truly representative* | Same community as exposed cohort* | Medical records* | No | Study controls for one or more additional confounding factors* | Medical records* | No description | Complete follow up* | Average |
| **Ejigu (2019)** | Truly representative* | Same community as exposed cohort* | Medical records* | No | Study controls for one or more additional confounding factors* | Medical records* | LNMP, symphysis-fundal height, or ultrasound (unspecified) | Complete follow up* | Average |
| **Ezechi (2012)** | Somewhat representative | Same community as exposed cohort* | Medical records* | No | Study controls for one or more additional confounding factors* | Medical records* | LNMP | <20% lost to follow up* | Average |
| **Favarato (2018)** | Truly representative* | Same community as exposed cohort* | No description | Yes* | Study controls for one or more additional confounding factors* | No description | No description | Complete follow up* | Poor |
| **Favarato (2019)** | Truly representative* | Same community as exposed cohort* | Medical records* | Yes* | Study controls for one or more additional confounding factors* | No description | No description | Complete follow up* | Poor |
| **Floridia (2020)** | Truly representative* | Same community as exposed cohort* | Medical records* | No | Study controls for one or more additional confounding factors* | Medical records* | Ultrasound, LNMP or both | Complete follow up* | Average |
| **Latham** | Truly representative* | Same community as exposed cohort* | Medical records* | No | Study controls for one or more additional confounding factors* | Medical records* | No description | <20% lost to follow up* | Poor |
| **Lopez (2015)** | Truly representative* | Same community as exposed cohort* | No description | Yes* | Study controls for one or more additional confounding factors* | No description | First trimester ultrasound and earliest available ultrasound in late gestation | Complete follow up* | Average |
| **Machado (2009)** | Somewhat representative | Same community as exposed cohort* | ART intake monitored as part of study* | Yes* | Study controls for one or more additional confounding factors* | No description | LNMP or ultrasound | Complete follow up* | Poor |
| **Patel (2022)** | Truly representative* | Same community as exposed cohort* | ART intake monitored as part of study* | No | Study controls for one or more additional confounding factors* | Medical records* | No description | <20% lost to follow up* | Average |
| **Piske (2021)** | Truly representative* | Same community as exposed cohort* | Medical records* | No | Study controls for one or more additional confounding factors* | Medical records* | LNMP | <20% lost to follow up* | Average |
| **Shapiro (2010)** | Truly representative* | Same community as exposed cohort* | ART intake monitored as part of study* | Yes* | Confounding factors not controlled for | Clinical observation* | LNMP, ultrasound (in 1st, 2nd, 3rd trimester) | Complete follow up* | Poor |
| **Short (2014)** | Truly representative* | Same community as exposed cohort* | Medical records* | No | Confounding factors not controlled for | Medical records* | No description | Complete follow up* | Poor |
| **Sibiude (2018)** | Truly representative* | Same community as exposed cohort* | Medical records* | No | Study controls for one or more additional confounding factors* | Unspecified | No description | Complete follow up* | Poor |
| **Sibiude (2021)** | Truly representative* | Same community as exposed cohort* | ART intake monitored as part of study* | Yes* | Study controls for one or more additional confounding factors* | Medical records* | LNMP confirmed by ultrasound | <20% lost to follow up* | Average |
| **Snijdewind (2018)** | Truly representative* | Same community as exposed cohort* | Medical records* | No | Study controls for one or more additional confounding factors* | Medical records* | Early ultrasound or LNMP | Complete follow up* | Average |
| **Szyld (2006)** | Truly representative* | Same community as exposed cohort* | ART intake monitored as part of study* | Yes* | Study controls for one or more additional confounding factors* | Clinical observation* | Other method:  LNMP with/without ultrasound, neonatal assessment (unspecified) | Complete follow up* | Average |
| **Townsend (2007)** | Truly representative* | Same community as exposed cohort* | No description | Yes* | Study controls for one or more additional confounding factors* | No description | No description | <20% lost to follow up* | Poor |
| **Van der Merwe (2011)** | Somewhat representative | Different source than the exposed cohort | Medical records* | No | Study controls for one or more additional confounding factors* | Medical records* | LNMP, ultrasound (unspecified), symphysis-fundal height, neonatal assessment (unspecified) | Complete follow up* | Poor |
| **Watts (2013)** | No description | Same community as exposed cohort* | Medical records* | No | Study controls for one or more additional confounding factors* | Medical records* | Clinical method (unspecified) and ultrasound (unspecified) | <20% lost to follow up* | Average |
| **Zash (2017)** | Truly representative* | Same community as exposed cohort* | Medical records* | No | Study controls for one or more additional confounding factors* | Medical records* | LNMP confirmed by ultrasound where possible | Complete follow up* | Average |
| **Zash (2017)** | Truly representative* | Same community as exposed cohort* | Medical records* | No | Study controls for one or more additional confounding factors* | Medical records* | LNMP confirmed by ultrasound where possible | Complete follow up* | Average |
| **Zash (2018)** | Truly representative* | Same community as exposed cohort* | Medical records* | No | Study controls for one or more additional confounding factors* | Medical records* | LNMP and/or ultrasound (unspecified), or symphysis-fundal height | Complete follow up* | Average |
| **Zash (2019)** | Truly representative* | Same community as exposed cohort* | Medical records* | No | Study controls for one or more additional confounding factors* | Clinical observation* | LNMP | <20% lost to follow up* | Average |
| **Zash (2021)** | Truly representative* | Same community as exposed cohort* | Medical records* | No | Confounding factors not controlled for | Medical records* | LNMP | <20% lost to follow up* | Poor |

Abbreviations: ART= antiretroviral therapy, BMI= body mass index, LNMP= last normal menstrual period.

## **Appendix 2.4**

## **Confounding factors adjusted for in included studies**

| **Study** | **Methods to correct for confounding factors** | **Regression analysis**  Confounders corrected for: | **Risk factor analysis**  Risk factors not significantly different between groups: | **Matching** |
| --- | --- | --- | --- | --- |
| **Aaron (2012)** | Regression analysis | Race, Smoking, Education, first viral load > 1000, First CD4 > 200, NNRTI | Not conducted for ART initiation groups analysed | - |
| **Albert (2020)** | Risk factor analysis | - | Maternal age, gravidity, previous PTB, time since HIV diagnosis | - |
| **Bailey (2019)** | Regression analysis | Adjusted a priori for potential confounders (for PTD models: calendar year and country of delivery, parity, maternal IDU history, CD4þ cell count, maternal age, third agent in the ART regimen; for SGA model: all variables included in the PTD models and infant sex and ART duration). | No p values reported | - |
| **Benamor Teixeira (2020)** | Risk factor analysis | - | Maternal age, ethnicity, marital status, syphilis, CD4 count, viral load | - |
| **Chauhan (2021)** | - | - | - | - |
| **Chen (2012)** | Regression analysis, risk factor analysis | CD4 count in pregnancy, advanced maternal age, nulliparity, maternal hypertension in pregnancy and anaemia | Nationality, education, parity, antenatal care received, syphilis, alcohol, smoking, CD4 count | - |
| **Delicio (2018)** | Risk factor analysis | - | Maternal age, co-infection, smoking, substance abuse, alcohol, CDC classification, ART start before or during pregnancy, duration of ART use, mode of delivery | - |
| **Ejigu (2019)** | Regression analysis, risk factor analysis | Maternal age, weight, marital status, education, parity, CD4 cell count during pregnancy, WHO clinical stage during pregnancy and timing of treatment initiation. | Marital status, CD4 count, haemoglobin | - |
| **Ezechi (2012)** | Regression analysis, risk factor analysis | Low birthweight, stage of HIV disease, reproductive tract infection and medical disorder. | Maternal age, parity, working status, social class, viral load, BMI, induced abortion history, spontaneous abortion, history of preterm delivery, history operative delivery, medical disorders | - |
| **Favarato (2018)** | Regression analysis | ART class in pregnancy, delivery year, maternal age at delivery, maternal origin, parity, history of IDU, first antenatal CD4+ cell count | Not conducted for ART initiation groups analysed | - |
| **Favarato (2019)** | Risk factor analysis | - | Year of delivery, country of delivery, maternal age, maternal origin, timing of first antenatal appointment, history of IVDU, ART at conception, | - |
| **Floridia (2020)** | Risk factor analysis | - | CD4 count, BMI, origin, sexual route of infection, nulligravida, HCV antbody, HBV coinfected, history of STDs, smoking, total cholesterol, LDL and HDL cholesterol, ALT, hemoglobin, vaginal infections, anaemia | - |
| **Latham (2022)** | - | - | - | - |
| **Lopez (2015)** | Risk factor analysis | - | Maternal age, black ethnicity, low educational level, smoking during pregnancy, drug use during pregnancy, nulliparity, chronic hypertension, assisted reproduction technique, 1st trimester mean pulsatility inded of the uterine arteries, HIV diagnosis in pregnancy, HIV transmission - blood transfusion, months of HIV infection, previous opportunistic infection, CD4 cell count <200 cells/uL at 1st trimester, CD4 cell count at 1st trimester, CD4 cell count at delivery, viral load <50 copies/ml at 1st trimester, viral load <50 copies/ml at delivery. | - |
| **Machado (2009)** | Regression analysis and risk factor analysis | Median age, year of delivery, indication of ARV, viral load at entry, Viral load at deliver (copies/ml), multiparity, alcohol use, STD, symptomatic disease | CD4 cell count, previous preterm delivery or miscarriage, mode of delivery, hepatitis B co- infection, hepatitis C co- infection, tobacco use, illicit drugs, hypertension | - |
| **Patel (2022)** | Regression analysis | Age at conception, race and ethnic group, education, timing of maternal HIV infection diagnosis, trimester at the first prenatal care visit, timing of ART initiation, smoking, alcohol, IDU, STD | - | - |
| **Piske (2021)** | Regression analysis and matching | Maternal substance use, smoking | - | Infant age, sex and geocode |
| **Shapiro (2010)** | - | - | No P-value reported | - |
| **Short (2014)** | - | - | Not conducted for ART initiation groups analysed | - |
| **Sibiude (2018)** | Regression, risk factor analysis | NRTIs (Zidovudine, abacavir, tenofovir), maternal age, and geographical origin | CD4 count at delivery, Viral load at delivery, Hospitalisation, pre-eclampsia, gestational diabetes | - |
| **Sibiude (2021)** | Regression analysis, matching | Age, ethnicity, multiple pregnancy | - | Type of ART backbone, maternal age, geographic origin, centre, year of delivery, gestational age at ART initiation, number of foetuses (singleton vs twins) |
| **Snijdewind (2018)** | Risk factor analysis | - | Region of origin, smoking, alcohol, IDU, mode of delivery | - |
| **Syzld (2006)** | Regression, risk factor analysis | Most complex ART regimen received during pregnancy, hypertension, mode of delivery, diabetes, adjusted BMI | Country of residence, ART regimen complexity, maternal age, viral load, CD4 count, alcohol, tobacco, marijuana, cocaine, indication for ART, STIs, history of preterm birth, renl disease, diabetes, anaemia, BMI | - |
| **Townsend (2007)** | Regression analysis | Repeat pregnancies, IDU, ethnic origin, maternal age at delivery, clinical status, +/- CD4 count | Not conducted for ART initiation groups analysed | - |
| **Van der Merwe (2011)** | Regression analysis, risk factor analysis | CD4 cell count, maternal age, hypertension, infant PCR | Race, smoking, alcohol, WHO stage, hypertension, diabetes, syphilis, gravidity, previous miscarriage, mode of delivery, infant gender | - |
| **Watts (2013)** | Regression analysis | Black or African American race, annual household income <$20 000/year, cigarette smoking, maternal CD4+ T-cell count of <200 cells/mm^3^ at delivery | - | - |
| **Zash (2017)** | Regression analysis | Maternal age, gravidity, low educational attainment | - | - |
| **Zash (2018)** | Regression analysis | Maternal age, gravidity, education | - | - |
| **Zash (2019)** | Regression analysis | Maternal age, gravidity, and educational attainment | - | - |
| **Zash (2021)** | - | - | - | - |
|  |  |  |  |  |

Abbreviations: ALT = alanine transaminase, ART= antiretroviral therapy, ARV = anti-retroviral drugs, BMI= body mass index, CDC= Centers for Disease Control and Prevention, GBS= group B streptococcus, HAART= highly active antiretroviral therapy, HBV= hepatitis B virus, HCV= hepatitis C virus, HDL = high density lipoprotein, HIV= human immunodeficiency virus, IDU= illicit drug use, IVDU = intravenous drug use, NNRTI = non-nucleoside reverse transcriptase inhibitor , LDL = low density lipoprotein, PTB=preterm birth, PTD = preterm delivery, SGA = small for gestational age, STD= sexually transmitted disease.

# Appendix 3

**Random-effects meta-analyses of risk of perinatal outcomes associated with women living with HIV receiving different classes of ART.**

Forest plots showing random-effects meta-analyses of risk of perinatal outcomes associated with women living with HIV receiving NNRTI-ART vs PI-ART, INSTI-ART vs PI-ART, NRTI-ART vs PI-ART, NRTI-ART vs NNRTI-ART, NNRTI-ART vs INSTI-ART. Relative risk (RR) and 95% confidence intervals (CIs) of individual studies and summary estimates are shown. All forest plots are based on unadjusted outcome frequencies of perinatal outcomes according to class of ART exposure.

**Appendix 3.1**

**Women living with HIV receiving NNRTI-ART vs PI-ART**

**Figure 3.1.1 Preterm birth in women living with HIV receiving NNRTI-ART vs PI-ART**

**Figure 3.1.2 Very preterm birth in women living with HIV receiving NNRTI-ART vs PI-ART**


**Figure 3.1.3 Spontaneous preterm birth in women living with HIV receiving NNRTI-ART vs PI-ART**

**Figure 3.1.4 Low birthweight in women living with HIV receiving NNRTI-ART vs PI-ART**

******

**Figure 3.1.5 Very low birthweight in women living with HIV receiving NNRTI-ART vs PI-ART**

**Figure 3.1.6 Small for gestational age in women living with HIV receiving NNRTI-ART vs PI-ART**

**Figure 3.1.7 Very small for gestational age in women living with HIV receiving NNRTI-ART vs PI-ART**

**Figure 3.1.8 Stillbirth and Neonatal death in women living with HIV receiving NNRTI-ART vs PI-ART**

| Outcome | Study | RR | 95% CI | P-value | NNRTI-ART | PI-ART |
| --- | --- | --- | --- | --- | --- | --- |
| SB | Favarato et al (2019) | 1.04 | 0.60, 1.79 | 0.891 | 19/2259 | 41/4693 |
| NND | Zash et al (2017) | 1.82 | 0.97, 3.40 | 0.063 | 70/4597 | 11/398 |

**Appendix 3.2**

**Women living with HIV receiving INSTI-ART vs PI-ART**

**Figure 3.2.1 Preterm birth in women living with HIV receiving INSTI-ART vs PI-ART**

**Figure 3.2.2 Very preterm birth in women living with HIV receiving INSTI-ART vs PI-ART**

**Figure 3.*2*.3 Low birthweight in women living with HIV receiving INSTI-ART vs PI-ART**

**Figure 3.2.4 Very low birthweight in women living with HIV receiving INSTI-ART vs PI-ART**

**Figure 3.*2*.5 Small for gestational age in women living with HIV receiving INSTI-ART vs PI-ART**

**Figure 3.2.6 Spontaneous Preterm Birth and Very small for gestational age in women living with HIV receiving INSTI-ART vs PI-ART**

| Outcome | Study | RR | 95% CI | P-value | INSTI-ART | PI-ART |
| --- | --- | --- | --- | --- | --- | --- |
| sPTB | Albert et al (2020) | 2.66 | 0.39, 18.18 | 0.391 | 1/18 | 48/325 |
| VSGA | Sibiude et al (2018) | 1.93 | 0.27, 13.66 | 0.508 | 1/48 | 62/1539 |

**Appendix 3.3**

**Women living with HIV receiving NRTI-ART vs PI-ART**

**Figure 3.3.1 Preterm birth in women living with HIV receiving NRTI-ART vs PI-ART**

**Figure 3.3.2 Spontaneous preterm birth in women living with HIV receiving NRTI-ART vs PI-ART**

**Figure 3.3.3 Small for gestational age in women living with HIV receiving NRTI-ART vs PI-ART**

**Figure 3.3.4 Very small for gestational age in women living with HIV receiving NRTI-ART vs PI-ART**

| Outcome | Study | RR | 95% CI | P-value | NRTI-ART | PI-ART |
| --- | --- | --- | --- | --- | --- | --- |
| VSGA | Aaron et al (2012) | 0.98 | 0.36, 2.68 | 0.970 | 4/27 | 17/117 |

**Appendix 3.4**

**Women living with HIV receiving NRTI-ART vs NNRTI-ART**

**Figure 3.4.1 Preterm birth in women living with HIV receiving NRTI-ART vs NNRTI-ART**

**Figure 3.4.2 Small for gestational age in women living with HIV receiving NRTI-ART vs NNRTI-ART**

**Figure 3.4.3 Very Preterm Birth, Spontaneous Preterm Birth, Low Birth Weight, Very Low Birth Weight, Very Small for Gestational Age in women living with HIV receiving NRTI-ART vs NNRTI-ART**

| Outcome | Study | RR | 95% CI | P-value | NRTI-ART | NNRTI-ART |
| --- | --- | --- | --- | --- | --- | --- |
| VPTB | Shapiro et al (2010) | 0.91 | 0.17, 4.90 | 0.910 | 4/283 | 2/156 |
| sPTB | Watts et al (2013) | 1.58 | 0.79, 3.15 | 0.196 | 13/193 | 17/160 |
| LBW | Shapiro et al (2010) | 1.13 | 0.70, 1.83 | 0.625 | 37/283 | 23/156 |
| VLBW | Shapiro et al (2010) | 1.36 | 0.31, 6.00 | 0.684 | 4/283 | 3/156 |
| VSGA | Aaron et al (2012) | 0.35 | 0.07, 1.76 | 0.201 | 4/27 | 2/39 |

**Appendix 3.5**

**Women living with HIV receiving NNRTI-ART vs INSTI-ART**

**Figure 3.5.1 Preterm birth in women living with HIV receiving NNRTI-ART vs INSTI-ART**

**Figure 3.5.2 Very preterm birth in women living with HIV receiving NNRTI-ART vs INSTI-ART**

**Figure 3.5.3 Low birthweight in women living with HIV receiving NNRTI-ART vs INSTI-ART**

**Figure 3.5.4 Very low birthweight in women living with HIV receiving NNRTI-ART vs INSTI-ART**

**Figure 3.5.5 Small for gestational age in women living with HIV receiving NNRTI-ART vs INSTI-ART**

**Figure 3.5.6 Very small for gestational age in women living with HIV receiving NNRTI-ART vs INSTI-ART**

**Figure 3.5.7 Neonatal death in women living with HIV receiving NNRTI-ART vs INSTI-ART**

**Figure 3.5.8 Spontaneous Preterm Birth in women living with HIV receiving NNRTI-ART vs INSTI-ART**

| Outcome | Study | RR | 95% CI | P-value | NNRTI-ART | INSTI-ART |
| --- | --- | --- | --- | --- | --- | --- |
| sPTB | Albert et al (2020) | 0.36 | 0.05, 2.60 | 0.312 | 12/78 | 1/18 |

# Appendix 4

# Subgroup analyses

**Appendix 4.1**

**Subgroup analysis by country income status**

| **Subgroup analyses by income status** | **Perinatal outcomes** | | | | | | | | |
| --- | --- | --- | --- | --- | --- | --- | --- | --- | --- |
|  | **PTB** | **VPTB** | **sPTB** | **LBW** | **VLBW** | **SGA** | **VSGA** | **Stillbirth** | **NND** |
|  | RR (95% CI)  **(**p-value) | RR (95% CI)  (p-value) | RR (95% CI)  (p-value) | RR (95% CI)  (p-value) | RR (95% CI)  (p-value) | RR (95% CI)  (p-value) | RR (95% CI)  (p-value) | RR (95% CI)  (p-value) | RR (95% CI)  (p-value) |
| **NNRTI-ART vs PI-ART** | | | | | | | | | |
| **High income countries** | 1.02  (0.88, 1.18)  (p=0.786) | 0.922  (0.35, 2.43)  (p=0.869) | 0.97  (0.67, 1.40)  (p=0.873) | 0.90  (0.73, 1.12)  (p=0.349) | 0.58  (0.34, 0.96)  (p=0.034) | 1.20  (1.03, 1.40)  (p=0.180) | 2.83  (0.69, 11.72)  (p=0.150) | 1.04  (0.60, 1.79)  (p=0.891) |  |
| **Low- and Middle- income countries** | 1.17  (0.87, 1.56)  (p=0.298) | 1.46  (1.00, 2.12)  (p=0.048) | 5.02  (3.62, 6.98)  (p<0.001) | 1.10  (0.79, 1.52)  (p=0.576) | 0.76  (0.08, 7.66)  (p=0.815) | 1.34  (0.89, 2.02)  (p=0.157) | 1.37  (1.05, 1.80)  (p=0.020) |  | 1.82  (0.97, 3.40)  (p=0.063) |
| **INSTI-ART vs PI-ART** | | | | | | | | | |
| **High income countries** | 0.97  (0.81, 1.18)  (p=0.784) | 0.85  (0.36, 2.01)  (p=0.714) | 2.66  (0.39, 18.18)  (p=0.319) | 1.17  (0.70, 1.95)  (p=0.548) | 0.58  (0.26, 1.31)  (p=0.190) | 1.02  (0.72, 1.43)  (p=0.916) | 1.93  (0.27, 13.66)  (p=0.508) |  |  |
| **Low- and Middle- income countries** | 1.98  (0.81, 4.84)  (p=0.136) |  |  | 2.67  (1.03, 6.91)  (p=0.043) |  | 1.06  (0.48, 2.38)  (p=0.879) |  |  |  |
| **NRTI-ART vs PI-ART** | | | | | | | | | |
| **High income countries** | 1.17  (0.82, 1.68)  (p=0.379) |  | 1.54  (0.89, 2.67)  (p=0.122) |  |  | 0.98  (0.66, 1.44)  (p=0.906) | 0.98 (0.36, 2.68)  (p=0.970) |  |  |
| **Low- and Middle- income countries** |  |  |  |  |  |  |  |  |  |
| **NRTI-ART vs NNRTI-ART** | | | | | | | | | |
| **High income countries** | 1.19  (0.84, 1.68)  (p=0.355) |  | 1.58  (0.79, 3.15)  (p=0.196) |  |  | 0.71  (0.28, 1.76)  (p=0.454) | 0.35  (0.07, 1.76)  (p=0.201) |  |  |
| **Low- and Middle- income countries** | 0.69  (0.40, 1.19)  (p=0.181) | 0.91  (0.17, 4.90)  (p=0.910) |  | 1.13  (0.70, 1.83)  (p=0.625) | 1.36  (0.31, 6.00)  (p=0.684) |  |  |  |  |
| **NNRTI-ART vs INSTI-ART** | | | | | | | | | |
| **High income countries** | 1.04  (0.64, 1.69)  (p=0.872) | 3.63  (0.37, 36.08)  (p=0.270) | 0.36  (0.05, 2.60)  (p=0.312) | 0.94  (0.42, 2.11)  (p=0.833) | 2.88  (0.19, 42.90)  (p=0.443) | 1.30  (0.73, 2.34)  (p=0.372) |  |  |  |
| **Low- and Middle- income countries** | 0.97  (0.89, 1.06)  (p=0.522) | 0.97  (0.80, 1.19)  (p=0.789) |  | 0.50  (0.20, 1.27)  (p=0.143) |  | 0.94  (0.86, 1.04)  (p=0.229) | 0.92  (0.80, 1.05)  (p=0.223) |  | 0.82  (0.56, 1.19)  (p=0.288) |

A RR > 1 indicates increased risk of a perinatal outcome associated with the second-mentioned ART class. For example, PI-ART is associated with an increased risk of very small for gestational age compared to NNRTI-ART in low- and middle- income countries (RR 1.37 95% CI 1.05, 1.80, p=0.020).

Abbreviations: ART= antiretroviral therapy, HIV= human immunodeficiency virus, PI = protease inhibitor, NNRTI = non-nucleoside reverse transcriptase inhibitor, NRTI = nucleoside reverse transcriptase inhibitor, INSTI = integrase strand transfer inhibitor, LBW= low birthweight, NND= neonatal death, PTB= preterm birth, RR= risk ratio, SGA= small for gestational age, sPTB= spontaneous preterm birth, VLBW= very low birthweight, VPTB= very preterm birth, VSGA= very small for gestational age.

## **Appendix 4.2**

**Number of studies and women analysed: country income status**

| **Perinatal outcomes** | | | | | | | | | |
| --- | --- | --- | --- | --- | --- | --- | --- | --- | --- |
|  | **PTB** | **VPTB** | **sPTB** | **LBW** | **VLBW** | **SGA** | **VSGA** | **Stillbirth** | **NND** |
|  | Number of studies  (number of women analysed) | Number of studies  (number of women analysed) | Number of studies  (number of women analysed) | Number of studies  (number of women analysed) | Number of studies  (number of women analysed) | Number of studies  (number of women analysed) | Number of studies  (number of women analysed) | Number of studies  (number of women analysed) | Number of studies  (number of women analysed) |
| **NNRTI-ART vs PI-ART** | | | | | | | | | |
| **High income countries** | 9  (20387) | 2  (2109) | 2  (1882) | 3  (2184) | 2  (2087) | 7  (16799) | 1  (156) | 1  (6952) |  |
| **Low- and Middle- income countries** | 9  (12496) | 2  (5421) | 1  (847) | 8  (4686) | 2  (1221) | 4  (7456) | 1  (4995) |  | 1  (4995) |
| **INSTI-ART vs PI-ART** | | | | | | | | | |
| **High income countries** | 4  (3191) | 2  (1685) | 1  (343) | 3  (1815) | 2  (1654) | 2  (1621) | 1  (1587) |  |  |
| **Low- and Middle- income countries** | 1  (202) |  |  | 1  (202) |  | 1  (156) |  |  |  |
| **NRTI-ART vs PI-ART** | | | | | | | | | |
| **High income countries** | 4  (3222) |  | 1  (1512) |  |  | 2  (1647) | 1  (144) |  |  |
| **Low- and Middle- income countries** |  |  |  |  |  |  |  |  |  |
| **NRTI-ART vs NNRTI-ART** | | | | | | | | | |
| **High income countries** | 4  (2507) |  | 1  (353) |  |  | 2  (417) | 1  (66) |  |  |
| **Low- and Middle- income countries** | 1  (439) | 1  (439) |  | 1  (439) | 1  (439) |  |  |  |  |
| **NNRTI-ART vs INSTI-ART** | | | | | | | | | |
| **High income countries** | 3  (655) | 2  (520) | 1  (96) | 3  (657) | 2  (517) | 2  (500) |  |  |  |
| **Low- and Middle- income countries** | 3  (12032) | 2  (15459) |  | 1  (274) |  | 3  (12082) | 2  (15519) |  | 2  (11756) |

Abbreviations: ART= antiretroviral therapy, HIV= human immunodeficiency virus, PI = protease inhibitor, NNRTI = non-nucleoside reverse transcriptase inhibitor, NRTI = nucleoside reverse transcriptase inhibitor, INSTI = integrase strand transfer inhibitor, LBW= low birthweight, NND= neonatal death, PTB= preterm birth, SGA= small for gestational age, sPTB= spontaneous preterm birth, VLBW= very low birthweight, VPTB= very preterm birth, VSGA= very small for gestational age.

**Appendix 4.3**

**I^2^ values: country income status**

|  | **Perinatal outcomes** | | | | | | | | |
| --- | --- | --- | --- | --- | --- | --- | --- | --- | --- |
|  | **PTB** | **VPTB** | **sPTB** | **LBW** | **VLBW** | **SGA** | **VSGA** | **Stillbirth** | **NND** |
|  | I^2^  (95% CI)  (p-value) | I^2^  (95% CI)  (p-value) | I^2^  (95%CI)  (p-value) | I^2^  (95% CI)  (p-value) | I^2^  (95% CI)  (p-value) | I^2^  (95% CI)  (p-value) | I^2^  (95% CI) (p-value) | I^2^  (95% CI) (p-value) | I^2^  (95%CI)  (p-value) |
| **NNRTI-ART vs PI-ART** | | | | | | | | | |
| **High income countries** | 47.0  (0.0, 77.0)  (p=0.057) | 25.0  (0.0, 85.1))  (p=0.248) | 0.00  (0.0, 0.0)  (p=0.962) | 0.00  (0.0, 53.5)  (p=0.615) | 0.00  (0.0, 65.5)  (p=0.448) | 32.0  (0.0, 73.1)  (p=0.184) | N/A | N/A |  |
| **Low- and Middle- income countries** | 81.3 (0.0, 92.8)  (p=0.000) | 0.0  (0.0, 0.0)  (p=0.543) | NA | 68.5  (0.0, 87.2)  (p=0.002) | 68.5  (0.0, 93.7)  (p=0.075) | 80.8 (0.0, 94.8)  (p=0.001) | N/A |  | N/A |
| **INSTI-ART vs PI-ART** | | | | | | | | | |
| **High income countries** | 0.0  (0.0, 52.6)  (p=0.604) | 0.0  (0.0, 57.6  (p=0.493) | N/A | 49.0  (0.0, 86.8)  (p=0.141) | 0.0  (0.0-63.4)  (p=0.461) | 0.0  (0.0, 41.7)  (p=0.559) | N/A |  |  |
| **Low- and Middle- income countries** | N/A |  |  | N/A |  | N/A |  |  |  |
| **NRTI-ART vs PI-ART** | | | | | | | | | |
| **High income countries** | 20.6  (0.0, 74.8)  (p=0.287) |  | N/A |  |  | 0.0  (0.0, 79.9)  (p=0.320) | N/A |  |  |
| **Low- and Middle- income countries** |  |  |  |  |  |  |  |  |  |
| **NRTI-ART vs NNRTI-ART** | | | | | | | | | |
| **High income countries** | 0.0  (0.0, 40.5)  (p=0.776) |  | N/A |  |  | 61.6  (0.0, 92.4)  (p=0.106) | N/A |  |  |
| **Low- and Middle- income countries** | N/A | N/A |  | N/A | N/A |  |  |  |  |
| **NNRTI-ART vs INSTI-ART** | | | | | | | | | |
| **High income countries** | 30.1  (0, 81.1)  (p=0.239) | 33.5  (0.0, 86.8)  (p=0.220) | NA | 61.8  (0.0, 90.1)  (p=0.073) | 68.4  (0.0, 93.7)  (p=0.075) | 0.0  (0.0,71.8)  (p=0.401) |  |  |  |
| **Low- and Middle- income countries** | 0.0  (0.0, 0.0)  (p=0.955) | 10.7  (0.0, 82.2)  (p=0.290) |  | N/A |  | 3.6  (0.0, 73.9)  (p=0.354) | 0.0  (0.0, 0.0)  (p=0.944) |  | 0.0  (0.0, 64.1)  (p=0.457) |

Abbreviations: ART= antiretroviral therapy, HIV= human immunodeficiency virus, PI = protease inhibitor, NNRTI = non-nucleoside reverse transcriptase inhibitor, NRTI = nucleoside reverse transcriptase inhibitor, INSTI = integrase strand transfer inhibitor, LBW= low birthweight, NND= neonatal death, PTB= preterm birth, SGA= small for gestational age, sPTB= spontaneous preterm birth, VLBW= very low birthweight, VPTB= very preterm birth, VSGA= very small for gestational age.

**Appendix 4.4**

**Subgroup analysis by country income status: test of subgroup difference**

| **Subgroup analyses by income status** | **Perinatal outcomes** | | | | | | | | |
| --- | --- | --- | --- | --- | --- | --- | --- | --- | --- |
|  | **PTB** | **VPTB** | **sPTB** | **LBW** | **VLBW** | **SGA** | **VSGA** | **Stillbirth** | **NND** |
|  | p-value | p-value | p-value | p-value | p-value | p-value | p-value | p-value | p-value |
| **NNRTI-ART vs PI-ART** | | | | | | | | | |
| **High income vs low/middle income** | 0.332 | 0.413 | 0.338 | 0.671 | 0.335 | 0.620 | 0.332 |  |  |
| **INSTI-ART vs PI-ART** | | | | | | | | | |
| **High income vs low/middle income** | 0.941 |  |  | 0.211 |  | 0.408 |  |  |  |
| **NRTI-ART vs PI-ART** | | | | | | | | | |
| **High income vs low/middle income** |  |  |  |  |  |  |  |  |  |
| **NRTI-ART vs NNRTI-ART** | | | | | | | | | |
| **High income vs low/middle income** | 0.931 |  |  |  |  |  |  |  |  |
| **NNRTI-ART vs INSTI-ART** | | | | | | | | | |
| **High income vs low/middle income** | 0.594 | 0.879 |  | 0.551 |  | 0.268 |  |  |  |

Test of subgroup differences by country income using a random-effects DerSimonian-Laird model. Abbreviations: ART= antiretroviral therapy, PI = protease inhibitor, NNRTI = non-nucleoside reverse transcriptase inhibitor, NRTI = nucleoside reverse transcriptase inhibitor, INSTI = integrase strand transfer inhibitor, LBW= low birthweight, NND= neonatal death, PTB= preterm birth, SGA= small for gestational age, sPTB= spontaneous preterm birth, VLBW= very low birthweight, VPTB= very preterm birth, VSGA= very small for gestational age.

**Appendix 4.5**

**Subgroup analysis by study quality**

| **Subgroup analyses by income status** | **Perinatal outcomes** | | | | | | | | |
| --- | --- | --- | --- | --- | --- | --- | --- | --- | --- |
|  | **PTB** | **VPTB** | **sPTB** | **LBW** | **VLBW** | **SGA** | **VSGA** | **Stillbirth** | **NND** |
|  | RR  (95% CI)  (p-value) | RR  (95% CI)  (p-value) | RR  (95% CI)  (p-value) | RR  (95% CI)  (p-value) | RR  (95% CI)  (p-value) | RR  (95% CI)  (p-value) | RR  (95% CI)  (p-value) | RR  (95% CI)  (p-value) | RR  (95% CI)  (p-value) |
| **NNRTI-ART vs PI-ART** | | | | | | | | | |
| **Average quality studies** | 1.07  (0.94, 1.21)  (p=0.296) | 1.14  (0.66, 2.00)  (p=0.635) | 1.70  (0.45, 6.35)  (p=0.429) | 1.13  (0.83, 1.53)  (p=0.429) | 0.58  (0.34, 0.96)  (p=0.034) | 1.19  (1.01, 1.41)  (p=0.040) | 1.41  (1.08, 1.83)  (p=0.011) |  | 1.82  (0.97, 3.4)  (p=0.063) |
| **Poor**  **quality studies** | 1.09  (0.76, 1.57)  (p=0.636) | 2.31  (0.50, 10.75)  (p=0.285) |  | 0.92  (0.67, 1.26)  (p=0.611) | 0.76  (0.07, 7.69)  (p=0.815) | 1.51  (0.91, 2.49)  (p=0.107) |  | 1.04  (0.60, 1.79)  (p=0.891) |  |
| **INSTI-ART vs PI-ART** | | | | | | | | | |
| **Average quality studies** | 1.05  (0.83, 1.32)  (p=0.941) | 0.85  (0.36, 2.01)  (p=714) | 2.66  (0.39, 18.18)  (p=0.391) | 1.91  (0.76, 4.76)  (p=0.408) | 0.58  (0.26, 1.31)  (p=0.190) | 1.29  (0.71, 2.34)  (p=0.408) |  |  |  |
| **Poor**  **quality studies** | 0.80  (0.41, 1.56)  (p=0.504) |  |  | 0.94  (0.50, 1.76)  (p=0.854) |  |  | 1.93  (0.27, 13.66)  (p=0.508) |  |  |
| **NRTI-ART vs PI-ART** | | | | | | | | | |
| **Average quality studies** | 0.74  (0.41, 1.32)  (p=0.740) |  | 1.54  (0.89, 2.67)  (p=0.122) |  |  | 0.98  (0.66, 1.46)  (p=0.906) | 0.98  (0.36, 2.68)  (p=0.970) |  |  |
| **Poor**  **quality studies** | 1.38  (1.00, 1.91)  (p=0.050) |  |  |  |  |  |  |  |  |
| **NRTI-ART vs NNRTI-ART** | | | | | | | | | |
| **Average quality studies** | 1.09  (0.73, 1.61)  (p=0.680) |  | 1.58  (0.79, 3.15)  (p=0.196) |  |  | 0.87  (0.43, 1.78)  (p=0.454) | 0.35  (0.07, 1.76)  (p=0.201) |  |  |
| **Poor**  **quality studies** | 1.02  (0.52, 2.00)  (p=0.961) | 0.91  (0.17, 4.90)  (p=0.910) |  | 1.13  (0.70, 1.83)  (p=0.625) | 1.36  (0.31, 6.00)  (p=0.684) |  |  |  |  |
| **NNRTI-ART vs INSTI-ART** | | | | | | | | | |
| **Average quality studies** | 0.97  (0.90, 1.06)  (p=0.558) | 1.03  (0.74 1.42)  (p=0.879) | 0.36  (0.05, 2.60)  (p=0.312) | 0.62  (0.23, 1.72)  (p=0.356) | 2.88  (0.19, 43.09)  (p=0.443) | 0.95  (0.87, 1.04)  (p=0.223) | 0.92  (0.80, 1.05)  (p=0.223) |  | 0.82  (0.56, 1.19)  (p=0.288) |
| **Poor**  **quality studies** | 1.90  (0.55, 2.54)  (p=0.660) |  |  | 1.37  (0.60, 3.11)  (p=0.457) |  |  |  |  |  |

A RR > 1 indicates increased risk of a perinatal outcome associated with the second-mentioned ART class. For example, PI-ART is associated with an increased risk of small for gestational age compared to NNRTI-ART in average quality studies (RR 1.19, 95% CI 1.01-1.41, p=0.040). Abbreviations: ART= antiretroviral therapy, HIV= human immunodeficiency virus, PI = protease inhibitor, NNRTI = non-nucleoside reverse transcriptase inhibitor, NRTI = nucleoside reverse transcriptase inhibitor, INSTI = integrase strand transfer inhibitor, LBW= low birthweight, NND= neonatal death, PTB= preterm birth, SGA= small for gestational age, sPTB= spontaneous preterm birth, VLBW= very low birthweight, VPTB= very preterm birth, VSGA= very small for gestational age.

**Appendix 4.6**

**Number of studies and women analysed: quality of studies**

|  | **Perinatal outcomes** | | | | | | | | |
| --- | --- | --- | --- | --- | --- | --- | --- | --- | --- |
|  | **PTB** | **VPTB** | **sPTB** | **LBW** | **VLBW** | **SGA** | **VSGA** | **Stillbirth** | **NND** |
|  | Number of studies  (number of women analysed) | Number of studies  (number of women analysed) | Number of studies  (number of women analysed) | Number of studies  (number of women analysed) | Number of studies  (number of women analysed) | Number of studies  (number of women analysed) | Number of studies  (number of women analysed) | Number of studies  (number of women analysed) | Number of studies  (number of women analysed) |
| **NNRTI-ART vs PI-ART** | | | | | | | | | |
| **Average quality studies** | 11  (21842) | 3  (7104) | 3  (2729) | 6  (5160) | 2  (2087) | 9  (17470) | 2  (5151) |  | 1  (4995) |
| **Poor**  **quality studies** | 7  (11041) | 1  (426) |  | 5  (1710) | 2  (1221) | 2  (6785) |  | 1  (6952) |  |
| **INSTI-ART vs PI-ART** | | | | | | | | | |
| **Average quality studies** | 4  (2706) | 2  (1685) | 1  (343) | 3  (1856) | 2  (1654) | 3  (1810) |  |  |  |
| **Poor**  **quality studies** | 1  (154) |  |  | 1  (161) |  |  | 1  (1587) |  |  |
| **NRTI-ART vs PI-ART** | | | | | | | | | |
| **Average quality studies** | 1  (284) |  | 1  (1512) |  |  | 2  (1647) | 1  (144) |  |  |
| **Poor**  **quality studies** | 3  (2938) |  |  |  |  |  |  |  |  |
| **NRTI-ART vs NNRTI-ART** | | | | | | | | | |
| **Average quality studies** | 2  (465) |  | 1  (353) |  |  | 2  (417) | 1  (66) |  |  |
| **Poor**  **quality studies** | 3  (2481) | 1  (439) |  | 1  (439) | 1  (439) |  |  |  |  |
| **NNRTI-ART vs INSTI-ART** | | | | | | | | | |
| **Average quality studies** | 5  (12552) | 3  (6676) | 1  (96) | 3  (761) | 2  (517) | 5  (12582) | 2  (15519) |  | 2  (11756) |
| **Poor**  **quality studies** | 1  (135) |  |  | 1  (170) |  |  |  |  |  |

Abbreviations: ART= antiretroviral therapy, HIV= human immunodeficiency virus, PI = protease inhibitor, NNRTI = non-nucleoside reverse transcriptase inhibitor, NRTI = nucleoside reverse transcriptase inhibitor, INSTI = integrase strand transfer inhibitor, LBW= low birthweight, NND= neonatal death, PTB= preterm birth, SGA= small for gestational age, sPTB= spontaneous preterm birth, VLBW= very low birthweight, VPTB= very preterm birth, VSGA= very small for gestational age.

**Appendix 4.7**

**I^2^ values: quality of studies**

|  | **Perinatal outcomes** | | | | | | | | |
| --- | --- | --- | --- | --- | --- | --- | --- | --- | --- |
|  | **PTB** | **VPTB** | **sPTB** | **LBW** | **VLBW** | **SGA** | **VSGA** | **Stillbirth** | **NND** |
|  | I^2^  (95% CI)  (p value) | I^2^  (95% CI)  (p value) | I^2^  (95% CI)  (p value) | I^2^  (95% CI)  (p value) | I^2^  (95% CI)  (p value) | I^2^  (95% CI)  (p value) | I^2^  (95% CI)  (p value) | I^2^  (95% CI)  (p value) | I^2^  (95% CI)  (p value) |
| **NNRTI-ART vs PI-ART** | | | | | | | | | |
| **Average quality studies** | 43.0  (0.0, 68.4)  (p = 0.063) | 55.8  (0.0, 85.2)  (p = 0.133) | 95.3  (0.0, 98.8)  (p = 0.000) | 65.2  (0.0, 87.2)  (p = 0.013) | 0.0  (0.0, 65.2)  (p = 0.448) | 36.5  (0.0, 72.1)  (p = 0.127) | 0.0  (0.0, 79.3)  (p = 0.326) |  | N/A |
| **Poor**  **quality studies** | 80.7  (0.0, 95.7)  (p = 0.000) | N/A |  | 72.7  (0.0, 78.2)  (p = 0.001) | 67.7  (0.0, 93.7)  (p =0.078) | 90.0  (0.0, 98.3)  (p = 0.000) |  | N/A |  |
| **INSTI-ART vs PI-ART** | | | | | | | | | |
| **Average quality studies** | 18.2  (0.0, 65.4)  (p = 0.300) | 0.0  (0.0, 57.6)  (p=0.493) | N/A | 72.2  (0.0, 85.4)  (p = 0.028) | 0.0  (0.0, 63.4  (p=0.461) | 44.5  (0.0, 5.4)  (p=0.165) |  |  |  |
| **Poor**  **quality studies** | N/A |  |  | N/A |  |  | N/A |  |  |
| **NRTI-ART vs PI-ART** | | | | | | | | | |
| **Average quality studies** | N/A |  | N/A |  |  | 0.00  (0.0, 79.9)  (p=0.320) | N/A |  |  |
| **Poor**  **quality studies** | 0.0  (0.0, 51.5)  (p = 0.824) |  |  |  |  |  |  |  |  |
| **NRTI-ART vs NNRTI-ART** | | | | | | | | | |
| **Average quality studies** | 0.0  (0.0, 11.2)  (p = 0.636) |  | N/A |  |  | 61.6  (0.0, 92.4)  (p=0.106) | N/A |  |  |
| **Poor**  **quality studies** | 30.3  (0.0, 84.4)  (p = 0.187) | N/A |  | N/A | N/A |  |  |  |  |
| **NNRTI-ART vs INSTI-ART** | | | | | | | | | |
| **Average quality studies** | 0.0  (0.0, 55.9)  (p = 0.559) | 41.7  (0.0, 83.3)  (p=0.162) | N/A | 72.6  (0.0, 92.5)  (p = 0.026) | 68.4  (0.0, 93.7)  (p=0.075) | 0.0  (0.0, 63.5)  (p=0.415) | 0.0  (0.0, 0.0)  (p=0.944) |  | 0.0  (0.0, 64.1)  (p=0.457) |
| **Poor**  **quality studies** | N/A |  |  | N/A |  |  |  |  |  |

Abbreviations: ART= antiretroviral therapy, HIV= human immunodeficiency virus, PI = protease inhibitor, NNRTI = non-nucleoside reverse transcriptase inhibitor, NRTI = nucleoside reverse transcriptase inhibitor, INSTI = integrase strand transfer inhibitor, LBW= low birthweight, NND= neonatal death, PTB= preterm birth, SGA= small for gestational age, sPTB= spontaneous preterm birth, VLBW= very low birthweight, VPTB= very preterm birth, VSGA= very small for gestational age.

**Appendix 4.8**

**Subgroup analysis by study quality: test of subgroup difference (interaction)**

| **Subgroup analyses by income status** | **Perinatal outcomes** | | | | | | | | |
| --- | --- | --- | --- | --- | --- | --- | --- | --- | --- |
|  | **PTB** | **VPTB** | **sPTB** | **LBW** | **VLBW** | **SGA** | **VSGA** | **Stillbirth** | **NND** |
|  | p-value | p-value | p-value | p-value | p-value | p-value | p-value | p-value | p-value |
| **NNRTI-ART vs PI-ART** | | | | | | | | | |
| **Average quality vs poor quality** | 0.322 | 0.413 |  | 0.671 | 0.335 | 0.380 |  |  |  |
| **INSTI-ART vs PI-ART** | | | | | | | | | |
| **Average quality vs poor quality** | 0.941 |  |  | 0.211 |  |  |  |  |  |
| **NRTI-ART vs PI-ART** | | | | | | | | | |
| **Average quality vs poor quality** | 0.379 |  |  |  |  |  |  |  |  |
| **NRTI-ART vs NNRTI-ART** | | | | | | | | | |
| **Average quality vs poor quality** | 0.931 |  |  |  |  |  |  |  |  |
| **NNRTI-ART vs INSTI-ART** | | | | | | | | | |
| **Average quality vs poor quality** | 0.594 |  |  | 0.551 |  |  |  |  |  |

Test of subgroup differences by study quality using a random-effects DerSimonian-Laird model. Abbreviations: ART= antiretroviral therapy, PI = protease inhibitor, NNRTI = non-nucleoside reverse transcriptase inhibitor, NRTI = nucleoside reverse transcriptase inhibitor, INSTI = integrase strand transfer inhibitor, LBW= low birthweight, NND= neonatal death, PTB= preterm birth, SGA= small for gestational age, sPTB= spontaneous preterm birth, VLBW= very low birthweight, VPTB= very preterm birth, VSGA= very small for gestational age.

#

# Appendix 5

# Sensitivity analyses

##

## **Appendix 5.1**

## **Adjusting for confounders in individual studies comparing women living with HIV receiving PI-ART, NNRTI-ART, INSTI-ART and NRTI-ART for preterm birth, very preterm birth, low birthweight, small for gestational age, very small for gestational age, stillbirth and neonatal death.**

| **Study** | **ART exposure** | **Perinatal outcome** | | | | | | | | | | | | | |
| --- | --- | --- | --- | --- | --- | --- | --- | --- | --- | --- | --- | --- | --- | --- | --- |
|  |  | **PTB** | | **VPTB** | | **LBW** | | **SGA** | | **VSGA** | | **Stillbirth** | | **NND** | |
|  |  | OR/RR (95% CI) | aOR/aRR (95% CI) | OR/RR (95% CI) | aOR/aRR (95% CI) | OR/RR (95% CI) | aOR/aRR (95% CI) | OR/RR  (95% CI) | aOR/aRR  (95% CI) | OR/RR (95% CI) | aOR/aRR (95% CI) | OR/RR (95% CI) | aOR/aRR (95% CI) | OR/RR (95% CI) | aOR/aRR (95% CI) |
| **Bailey et al**  **(2019)**** | Third agent: Other PI | ref | ref |  |  |  |  | ref | ref |  |  |  |  |  |  |
|  | Third agent: NNRTI | 1.13  (0.89, 1.60) | 1.17  (0.81, 1.68) |  |  |  |  | 0.98  (0.66, 1.45) | 0.85  (0.65, 1.29) |  |  |  |  |  |  |
| **Ejigu et al**  **(2019)** | EFV-based ART | ref | ref |  |  | ref | ref | ref | ref |  |  |  |  |  |  |
|  | PI-based ART | 1.75  (0.77, 3.98) | 1.81  (0.78, 4.18) |  |  | 0.64  (0.18, 2.26) | 0.62  (0.17, 2.28) | 0.65  (0.26, 1.62) | 0.66  (0.25, 1.75) |  |  |  |  |  |  |
| **Favarato et al**  **(2018)** | NNRTI + 2NRTI | ref | ref |  |  |  |  |  |  |  |  |  |  |  |  |
|  | LPV/r + 2NRTI | 1.39  (1.13, 1.70) | 1.56  (1.19, 2.04) |  |  |  |  |  |  |  |  |  |  |  |  |
|  | Other PI/r + 2NRTI | 1.09  (0.88, 1.36) | 1.10  (0.84, 1.45) |  |  |  |  |  |  |  |  |  |  |  |  |
| **Zash et al (2017)** | TDF-FTC-EFV | ref | ref |  |  |  |  | ref | ref | ref | ref |  |  | ref | ref |
|  | TDF-FTC-LPV-R | 1.11  (0.87, 1.41) | 1.12  (0.88, 1.43) |  |  |  |  | 1.62  (1.29, 2.03) | 1.56  (1.25, 1.97) | 1.87  (1.31, 2.67) | 1.81  (1.26, 2.59) |  |  | 1.50  (0.53, 4.24) | 1.60  (0.56, 4.56) |
|  | ZDV-3TC-LPV-R | 1.36  (1.07, 1.74) | 1.36  (1.06, 1.75) |  |  |  |  | 1.19  (0.87, 1.63) | 1.13  (0.82, 1.56) | 1.75  (1.15, 2.67) | 1.70 (1.10, 2.62) |  |  | 3.64  (1.62, 8.17) | 4.01  (1.78, 9.11) |
| **Zash et al (2018)** | EFV based ART | ref | ref | ref | ref |  |  | ref | ref | ref | ref | ref | ref | ref | ref |
|  | (DTG) based ART | 0.97 (0.87, 1.10) | 0.98 (0.87, 1.11) | 1.10 (0.83, 1.45) | 1.09 (0.82, 1.45) |  |  | 0.94 (0.83, 1.06) | 0.94  (0.83, 1.06) | 0.91 (0.74, 1.13) | 0.91 (0.74, 1.13) | 0.99 (0.69, 1.42) | 0.99 (0.69, 1.42) | 0.93 (0.57, 1.53) | 0.96 (0.58, 1.57) |
| **Patel et al (2022)** | DTG-based ART | ref | ref |  |  | ref | ref | ref | ref |  |  |  |  |  |  |
|  | ATV/r-based ART | n.s | n.s |  |  | n.s | n.s | n.s | n.s |  |  |  |  |  |  |
|  | DRV/r-based ART | n.s | n.s |  |  | n.s | n.s | n.s | n.s |  |  |  |  |  |  |
|  | RPV-based ART | n.s | n.s |  |  | n.s | n.s | n.s | n.s |  |  |  |  |  |  |
|  | RAL-based ART | n.s | n.s |  |  | n.s | n.s | n.s | n.s |  |  |  |  |  |  |
|  | EVG/c-based ART | n.s | n.s |  |  | n.s | n.s | n.s | n.s |  |  |  |  |  |  |

n.s.= not statistically significant from the reference

Abbreviations: ART= antiretroviral therapy, HIV= human immunodeficiency virus, PI = protease inhibitor, NNRTI = non-nucleoside reverse transcriptase inhibitor, NRTI = nucleoside reverse transcriptase inhibitor, INSTI = integrase strand transfer inhibitor, LBW= low birthweight, NND= neonatal death, PTB= preterm birth, SGA= small for gestational age, sPTB= spontaneous preterm birth, VPTB= very preterm birth, VSGA= very small for gestational age.

**Appendix 6**

**Perinatal outcomes of women living with HIV receiving specific ‘third drugs’ of different ART classes.**

**Appendix 6.1. Number of studies and women analysed: comparisons of specific drugs from different classes.**

**Appendix 6.1A**

| **All studies** |  |  |  |  |  |  |  |  |  |
| --- | --- | --- | --- | --- | --- | --- | --- | --- | --- |
|  | **PTB** | **VPTB** | **sPTB** | **LBW** | **VLBW** | **SGA** | **VSGA** | **Stillbirth** | **NND** |
|  | Number of studies  (number of women analysed) | Number of studies  (number of women analysed) | Number of studies  (number of women analysed) | Number of studies  (number of women analysed) | Number of studies  (number of women analysed) | Number of studies  (number of women analysed) | Number of studies  (number of women analysed) | Number of studies  (number of women analysed) | Number of studies  (number of women analysed) |
| **NNRTI-ART vs PI-ART** | | | | | | | | | |
| ***NVP vs LPV/r*** | 5  (7090) | 2  (2949) |  | 3  (1666) | 2  (1106) | 2  (3155) | 1  (2523) | 1  (4387) | 1  (2523) |
| ***NVP vs ATV/r*** | 1  (162) |  |  | 2  (195) |  |  |  | 1  (2658) |  |
| ***NVP vs NFV*** | 1  (267) |  |  | 1  (270) |  |  |  |  |  |
| ***EFV vs LPV/r*** | 2  (3403) | 1  (2870) |  | 1  (534) | 1  (534) | 2  (3353) | 1  (2870) | 1  (3852) | 1  (2870) |
| ***EFV vs ATV/r*** |  |  |  | 1  (54) |  |  |  | 1  (2123) |  |
| ***EFV vs DRV/r*** |  |  |  |  |  |  |  | 1  (1304) |  |

**Appendix 6.1B**

| **All Studies** | **PTB** | **VPTB** | **sPTB** | **LBW** | **VLBW** | **SGA** | **VSGA** | **Stillbirth** | **NND** |
| --- | --- | --- | --- | --- | --- | --- | --- | --- | --- |
|  | Number of studies  (number of women analysed) | Number of studies  (number of women analysed) | Number of studies  (number of women analysed) | Number of studies  (number of women analysed) | Number of studies  (number of women analysed) | Number of studies  (number of women analysed) | Number of studies  (number of women analysed) | Number of studies  (number of women analysed) | Number of studies  (number of women analysed) |
| **INSTI-ART vs PI-ART** | | | | | | | | | |
| ***DTG vs ATV/r*** | 1  (584) | 1  (584) |  | 1  (584) | 1  (584) | 1  (584) |  |  |  |
| ***DTG vs DRV/r*** | 1  (305) | 1  (305) |  | 1  (305) | 1  (305) | 1  (305) |  |  |  |
| ***RAL vs LPV/r*** |  |  |  |  |  |  | 1  (763) |  |  |
| ***RAL vs ATV/r*** | 1  (550) | 1  (550) |  | 1  (550) | 1  (550) | 1  (550) | 1  (584) |  |  |
| ***RAL vs DRV/r*** | 1  (271) | 1  (271) |  | 1  (271) | 1  (271) | 1  (271) | 1  (336) |  |  |
| ***EVG/c vs ATV/r*** | 1  (623) | 1  (623) |  | 1  (623) | 1  (623) | 1  (623) |  |  |  |
| ***EVG/c vs DRV/r*** | 1  (344) | 1  (344) |  | 1  (344) | 1  (344) | 1  (344) |  |  |  |

**Appendix 6.1C**

| ***All Studies*** | **PTB** | **VPTB** | **sPTB** | **LBW** | **VLBW** | **SGA** | **VSGA** | **Stillbirth** | **NND** |
| --- | --- | --- | --- | --- | --- | --- | --- | --- | --- |
|  | Number of studies  (number of women analysed) | Number of studies  (number of women analysed) | Number of studies  (number of women analysed) | Number of studies  (number of women analysed) | Number of studies  (number of women analysed) | Number of studies  (number of women analysed) | Number of studies  (number of women analysed) | Number of studies  (number of women analysed) | Number of studies  (number of women analysed) |
| **NRTI-ART vs NNRTI-ART** | | | | | | | | | |
| ***ABC vs NVP*** | 1  (142) |  |  |  |  |  |  |  |  |

**Appendix 6.1D**

| ***All Studies*** | **PTB** | **VPTB** | **sPTB** | **LBW** | **VLBW** | **SGA** | **VSGA** | **Stillbirth** | **NND** |
| --- | --- | --- | --- | --- | --- | --- | --- | --- | --- |
|  | Number of studies  (number of women analysed) | Number of studies  (number of women analysed) | Number of studies  (number of women analysed) | Number of studies  (number of women analysed) | Number of studies  (number of women analysed) | Number of studies  (number of women analysed) | Number of studies  (number of women analysed) | Number of studies  (number of women analysed) | Number of studies  (number of women analysed) |
| **NNRTI-ART vs INSTI-ART** | | | | | | | | | |
| ***EFV vs DTG*** | 2  (11779) | 2  (15459) |  |  |  | 2  (11835) | 2  (15519) |  | 1  (6190) |
| ***EFV vs RAL*** | 1  (273) |  |  | 1  (274) |  | 1  (247) |  |  |  |
| ***RPV vs DTG*** | 1  (350) | 1  (350) |  | 1  (350) | 1  (350) | 1  (350) |  |  |  |

ART=antiretroviral therapy, INSTI=integrase strand transfer inhibitor, NNRTI=non-nucleoside reverse transcriptase inhibitor, NRTI=nucleoside reverse transcriptase inhibitor, PI=protease inhibitor.

Protease inhibitors: APV=amprenavir, ATV=atazanavir, DRV=darunavir, FPV=fosamprenavir, IDV=indinavir, LPV=lopinavir, NFV=nelfinavir, RTV=ritonavir, /r=ritonavir boosted, /c = cobicistat boosted.

Non-nucleoside reverse transcriptase inhibitors: EFV = efavirenz, NVP = nevirapine

Nucleoside reverse transcriptase inhibitors: ABC = abacavir.

Integrase strand transfer inhibitors: EVG = elvitegravir, DTG = dolutegravir, RAL=raltegravir, BIC = bictegravir.

Perinatal outcomes: LBW=low birthweight, PTB=preterm birth, SGA=small for gestational age, sPTB=spontaneous preterm birth, VLBW=very low birthweight, VPTB=very preterm birth, VSGA=very small for gestational age, NND = neonatal death.

**Appendix 6.2**

***I^2^* values: comparisons of specific drugs from different classes**

**Appendix 6.2A: NNRTI-ART vs PI-ART**

| **All studies** |  |  |  |  |  |  |  |  |  |
| --- | --- | --- | --- | --- | --- | --- | --- | --- | --- |
|  | **PTB** | **VPTB** | **sPTB** | **LBW** | **VLBW** | **SGA** | **VSGA** | **Stillbirth** | **NND** |
|  | I^2^  (95% CI)  (p value) | I^2^  (95% CI)  (p value) | I^2^  (95% CI)  (p value) | I^2^  (95% CI)  (p value) | I^2^  (95% CI)  (p value) | I^2^  (95% CI)  (p value) | I^2^  (95% CI)  (p value) | I^2^  (95% CI)  (p value) | I^2^  (95% CI)  (p value) |
| **NNRTI-ART vs PI-ART** | | | | | | | | | |
| ***NVP vs LPV/r*** | 87.4  (0.0, 96.0)  (0.000) | 0.0  (0.0, 68.4)  (0.424) |  | 29.6  (0.0, 0.0)  (0.242) | 78.9  (0.0, 95.4)  (0.030) | 96.3  (0.0, 99.3)  (0.000) | N/A | N/A | N/A |
| ***NVP vs ATV/r*** | N/A |  |  | 0.0  (0.0, 0.0)  (0.767) |  |  |  | N/A |  |
| ***NVP vs NFV*** | N/A |  |  | N/A |  |  |  |  |  |
| ***EFV vs LPV/r*** | 95.7  (0.0, 0.0)  (0.000) | N/A |  | N/A | N/A | N/A | N/A | N/A | N/A |
| ***EFV vs ATV/r*** |  |  |  | N/A |  |  |  | N/A |  |
| ***EFV vs DRV/r*** |  |  |  |  |  |  |  | N/A |  |

**Appendix 6.2B: INSTI-ART vs PI-ART**

| **All Studies** | **PTB** | **VPTB** | **sPTB** | **LBW** | **VLBW** | **SGA** | **VSGA** | **Stillbirth** | **NND** |
| --- | --- | --- | --- | --- | --- | --- | --- | --- | --- |
|  | I^2^  (95% CI)  (p value) | I^2^  (95% CI)  (p value) | I^2^  (95% CI)  (p value) | I^2^  (95% CI)  (p value) | I^2^  (95% CI)  (p value) | I^2^  (95% CI)  (p value) | I^2^  (95% CI)  (p value) | I^2^  (95% CI)  (p value) | I^2^  (95% CI)  (p value) |
| **INSTI-ART vs PI-ART** | | | | | | | | | |
| ***DTG vs ATV/r*** | N/A | N/A |  | N/A | N/A | N/A |  |  |  |
| ***DTG vs DRV/r*** | N/A | N/A |  | N/A | N/A | N/A |  |  |  |
| ***RAL vs LPV/r*** |  |  |  |  |  |  | N/A |  |  |
| ***RAL vs ATV/r*** | N/A | N/A |  | N/A | N/A | N/A | N/A |  |  |
| ***RAL vs DRV/r*** | N/A | N/A |  | N/A | N/A | N/A | N/A |  |  |
| ***EVG/c vs ATV/r*** | N/A | N/A |  | N/A | N/A | N/A |  |  |  |
| ***EVG/c vs DRV/r*** | N/A | N/A |  | N/A | N/A | N/A |  |  |  |

**Appendix 6.2C: NRTI-ART vs NNRTI-ART**

| **All Studies** | **PTB** | **VPTB** | **sPTB** | **LBW** | **VLBW** | **SGA** | **VSGA** | **Stillbirth** | **NND** |
| --- | --- | --- | --- | --- | --- | --- | --- | --- | --- |
|  | I^2^  (p value) | I^2^  (p value) | I^2^  (p value) | I^2^  (p value) | I^2^  (p value) | I^2^  (p value) | I^2^  (p value) | I^2^  (p value) | I^2^  (p value) |
| **NRTI-ART vs NNRTI-ART** | | | | | | | | | |
| ***ABC vs NVP*** | N/A |  |  |  |  |  |  |  |  |

**Appendix 6.2D: NNRTI-ART vs INSTI-ART**

| **All Studies** | **PTB** | **VPTB** | **sPTB** | **LBW** | **VLBW** | **SGA** | **VSGA** | **Stillbirth** | **NND** |
| --- | --- | --- | --- | --- | --- | --- | --- | --- | --- |
|  | I^2^  (95% CI)  (p value) | I^2^  (95% CI)  (p value) | I^2^  (95% CI)  (p value) | I^2^  (95% CI)  (p value) | I^2^  (95% CI)  (p value) | I^2^  (95% CI)  (p value) | I^2^  (95% CI)  (p value) | I^2^  (95% CI)  (p value) | I^2^  (95% CI)  (p value) |
| **NNRTI-ART vs INSTI-ART** | | | | | | | | | |
| ***EFV vs DTG*** | 0.0  (0.0, 0.0)  (0.855) | 10.7  (0.0, 82.2)  (0.290) |  |  |  | 0.0  (0.0, 0.0)  (0.935) | 0.0  (0.0, 0.0)  (0.944) |  | N/A |
| ***EFV vs RAL*** | N/A |  |  | N/A |  | N/A |  |  |  |
| ***RPV vs DTG*** | N/A | N/A |  | N/A | N/A | N/A |  |  |  |

ART=antiretroviral therapy, INSTI=integrase strand transfer inhibitor, NNRTI=non-nucleoside reverse transcriptase inhibitor, NRTI=nucleoside reverse transcriptase inhibitor, PI=protease inhibitor.

Protease inhibitors: APV=amprenavir, ATV=atazanavir, DRV=darunavir, FPV=fosamprenavir, IDV=indinavir, LPV=lopinavir, NFV=nelfinavir, RTV=ritonavir, /r=ritonavir boosted, /c = cobicistat boosted.

Non-nucleoside reverse transcriptase inhibitors: EFV = efavirenz, NVP = nevirapine

Nucleoside reverse transcriptase inhibitors: ABC = abacavir.

Integrase strand transfer inhibitors: EVG = elvitegravir, DTG = dolutegravir, RAL=raltegravir, BIC = bictegravir.

Perinatal outcomes: LBW=low birthweight, PTB=preterm birth, SGA=small for gestational age, sPTB=spontaneous preterm birth, VLBW=very low birthweight, VPTB=very preterm birth, VSGA=very small for gestational age, NND = neonatal death.

**Appendix 6.3 *I^2^* values: comparisons of different ART Classes**

| **All Studies** | **PTB** | **VPTB** | **sPTB** | **LBW** | **VLBW** | **SGA** | **VSGA** | **Stillbirth** | **NND** |
| --- | --- | --- | --- | --- | --- | --- | --- | --- | --- |
|  | *I^2^*  (95% CI)  (p value) | *I^2^*  (95% CI)  (p value) | *I^2^*  (95% CI)  (p value) | *I^2^*  (95% CI)  (p value) | *I^2^*  (95% CI)  (p value) | *I^2^*  (95% CI)  (p value) | *I^2^*  (95% CI)  (p value) | *I^2^*  (95% CI)  (p value) | *I^2^*  (95% CI)  (p value) |
| **NNRTI-ART vs PI-ART** | 71.2  (18.8, 85.4%)  (p=0.000) | 29.9  (0.0, 78.3)  (p=0.233) | 95.3  (0.0, 98.8)  (p=0.000) | 58.1  (0.0, 80.7)  (p=0.008) | 37.7  (0.0, 80.5)  (p=0.186) | 62.0  (0.0, 83.8)  (p=0.003) | 0.0  (0.0, 79.3)  (p=0.326) | NA | NA |
| **INSTI-ART vs PI-ART** | 3.6  (0.0, 65.4)  (p=0.386) | 0.0  (0.0, 57.6)  (p=0.493) | NA | 58.7  (0.0, 87.7)  (p=0.064) | 0.0  (0.0, 63.4)  (p=0.461) | 44.5  (0.0, 5.4)  (p=0.165) | NA |  |  |
| **NRTI-ART vs PI-ART** | 20.6  (0.0, 74.8)  (p=0.287) |  | NA |  |  | 0.00  (0.0, 79.9)  (p=0.320) | NA |  |  |
| **NRTI-ART vs NNRTI-ART** | 0.0  (0.0-62.4)  (p=0.432) | NA | NA | NA | NA | 61.6  (0.0, 92.4)  (p=0.106) | NA |  |  |
| **NNRTI-ART vs INSTI-ART** | 0.0  (0.0, 51.8)  (p=0.662) | 41.7  (0.0, 83.3)  (p=0.162) | NA | 61.0  (0.0, 87.9)  (p=0.053) | 68.4  (0.0, 93.7)  (p=0.075) | 0.0  (00, 63.5)  (p=0.415) | 0.0  (0.0, 0.0)  (p=0.944) |  | 0.0  (0.0, 64.1)  (p=0.457) |

Abbreviations: ART= antiretroviral therapy, HIV= human immunodeficiency virus, PI = protease inhibitor, NNRTI = non-nucleoside reverse transcriptase inhibitor, NRTI = nucleoside reverse transcriptase inhibitor, INSTI = integrase strand transfer inhibitor, LBW= low birthweight, NND= neonatal death, PTB= preterm birth, SGA= small for gestational age, sPTB= spontaneous preterm birth, VLBW= very low birthweight, VPTB= very preterm birth, VSGA= very small for gestational age.
